# Topical Magnesium Orotate Lipogel in Kidney Transplant Recipients with Persistent Hypomagnesemia: Formulation Development and Pilot Clinical Study

**DOI:** 10.3390/nu18162740

**Published:** 2026-08-21

**Authors:** Corina Moisa, Florin Bănică, Ioana Adela Rațiu, Octavia Gligor, Laura Grațiela Vicaș, Cristian Adrian Rațiu, Mădălin Florin Ganea, Csaba Nagy, Anamaria Ratiu, Edy Hagi Islai, Mariana Ganea

**Affiliations:** 1 Faculty of Medicine and Pharmacy, University of Oradea, 1st December Square 10, 410073 Oradea, Romania; cmoisa@uoradea.ro (C.M.); fbanica@uoradea.ro (F.B.); octavia.gligor@uoradea.ro (O.G.); cristian.ratiu@didactic.uoradea.ro (C.A.R.); ganea.madalinflorin@student.uoradea.ro (M.F.G.); mganea@uoradea.ro (M.G.); 2 Doctoral School of Biomedical Sciences, University of Oradea, 1 Universitătii Street, 410073 Oradea, Romania; nagy.csaba@student.uoradea.ro; 3 Faculty of Dentistry, University of Medicine and Pharmacy “Iuliu Hatieganu” Cluj-Napoca, Victor Babeș Street 8, 400347 Cluj-Napoca, Romania; ratiu.anamaria@elearn.umfcluj.ro (A.R.); hagi.islai.edy@elearn.umfcluj.ro (E.H.I.)

**Keywords:** magnesium deficiency, physical activity, kidney transplantation, orotate magnesium lipogel, topical preparation, quality of life

## Abstract

**Background**: In solid-organ transplant recipients, hypomagnesemia is a frequent and persistent complication, predominantly related to long-term use of calcineurin inhibitors. Oral magnesium supplementation is often ineffective due to poor adherence, gastrointestinal adverse effects, and high treatment costs. **Objectives**: This pilot study aimed to (i) develop a topical magnesium formulation for use in renal transplant recipients with persistent hypomagnesemia; (ii) evaluate the influence of formulation excipients on magnesium release from pharmaceutical preparations; and (iii) preliminarily assess the clinical outcomes, functional parameters, patient satisfaction, and tolerability associated with topical magnesium lipogel use in renal transplant recipients with persistent hypomagnesemia. **Materials and Methods**: Three lipogel formulations containing magnesium orotate, citrate, or sulfate were prepared and characterized in terms of organoleptic properties, pH, colloidal stability, and rheological behavior. In vitro magnesium release was evaluated using Franz diffusion cells. The formulation demonstrating the most favorable release profile, magnesium orotate lipogel, was administered twice daily for two months to 21 renal transplant recipients with persistent hypomagnesemia who had shown inadequate response, intolerance, or non-adherence to oral magnesium supplementation. Laboratory parameters were assessed at baseline and after treatment. Patient satisfaction was evaluated using the Lübeck questionnaire, while physical activity and exercise capacity were assessed using the International Physical Activity Questionnaire (IPAQ) and metabolic equivalent task (MET) calculations. **Results**: Franz cell studies demonstrated superior magnesium release from the magnesium orotate lipogel compared with the other formulations. After two months, serum magnesium levels were higher than baseline (1.554 ± 0.124 vs. 1.448 ± 0.111 mg/dL, *p* < 0.001), although they remained below the normal reference range. No significant changes were observed in eGFR or hsCRP. Moderate-intensity physical activity increased significantly (310.475 ± 92.505 vs. 279.286 ± 89.907 MET-min/week, *p* < 0.001), while vigorous-intensity activity did not change significantly. No relevant adverse effects were reported. Patient satisfaction was high across all evaluated domains, including practicability (86.6%), efficacy (96.4%), tolerability (97.1%), and trust in medical professionals (97.6%). **Conclusions**: In this pilot study, topical magnesium orotate lipogel was well tolerated and accepted by renal transplant recipients with persistent hypomagnesemia. Serum magnesium levels and moderate physical activity improved over the two-month observation period, although magnesium levels remained below the normal range. These preliminary findings warrant confirmation in larger, randomized, placebo-controlled studies.

## 1. Introduction

Magnesium (Mg) is a cation that plays a major role in all biochemical processes throughout the organism. It is a cofactor for more than 300 enzymes, among which Na+/K+-dependent-ATPase, which acts as a natural calcium channel blocker, as a consequence of maintaining potassium inside the cell. The majority of Mg is stored in bones and muscles, with intracellular concentrations approximately ten times higher than those in the extracellular compartment. In the intracellular compartment, along with adenosine triphosphate (ATP), Mg forms a complex involved in mitochondrial oxidative phosphorylation and in preventing free radical production [1,2]. Hypomagnesemia is associated with an insufficient dietary Mg intake due to industrial-scale use of pesticides [3]. Another significant factor contributing to low levels of Mg in the organism is represented by the administration of certain medications, which influence its metabolism. It is well established that Mg, calcium, and sodium levels are closely interrelated in the human body, with high dietary intake of calcium and sodium leading to increased Mg excretion [4]. A commonly used method for patients to maintain Mg levels within the normal range is taking Mg-containing supplements. However, it is important to note that not all Mg compounds exhibit the same bioavailability, as numerous factors can influence the achievement of an adequate therapeutic response [5,6].

In skeletal muscle, hypomagnesemia is associated with muscular cramps, spasms, inflammatory diseases, and neuromuscular disorders [7,8]. Watkins and Sircus have studied the transdermal absorption of Mg over 3 months in a small number of patients using Mg oil formulated as a spray for daily application. Results were encouraging as they indicated cellular Mg levels up to 262% [9,10]. Eisenkraft studied changes in serum Mg levels in healthy volunteers after repeated application of a Mg-containing lotion. Nevertheless, the results were not substantially different compared to the placebo group [11]. Mayuramas et al. did not obtain compelling evidence of an increase in cellular Mg levels following repeated application of several Mg chloride solutions. Nonetheless, positive results were obtained following the application of several Mg chloride-containing lipogels [12]. Increases in serum Mg of up to 9% were also observed following administration of a serum with Mg on the tegument of a group of 25 patients [13].

Franz cells are frequently used to evaluate the in vitro activity of semisolid preparations containing an active ingredient. It provides information regarding mass flux and release time of the active ingredient [14].

A distinct category of patients with a high prevalence of hypomagnesemia is represented by kidney transplant recipients. The administration of calcineurin inhibitors as first-line agents in post-transplant immunosuppressive therapy constitutes the principal cause of decreased serum magnesium levels in this population.

Although hypomagnesemia is nearly universal during the first year after transplantation, it persists long-term in a significant proportion of patients, regardless of tacrolimus trough levels.

**Figure 1:**
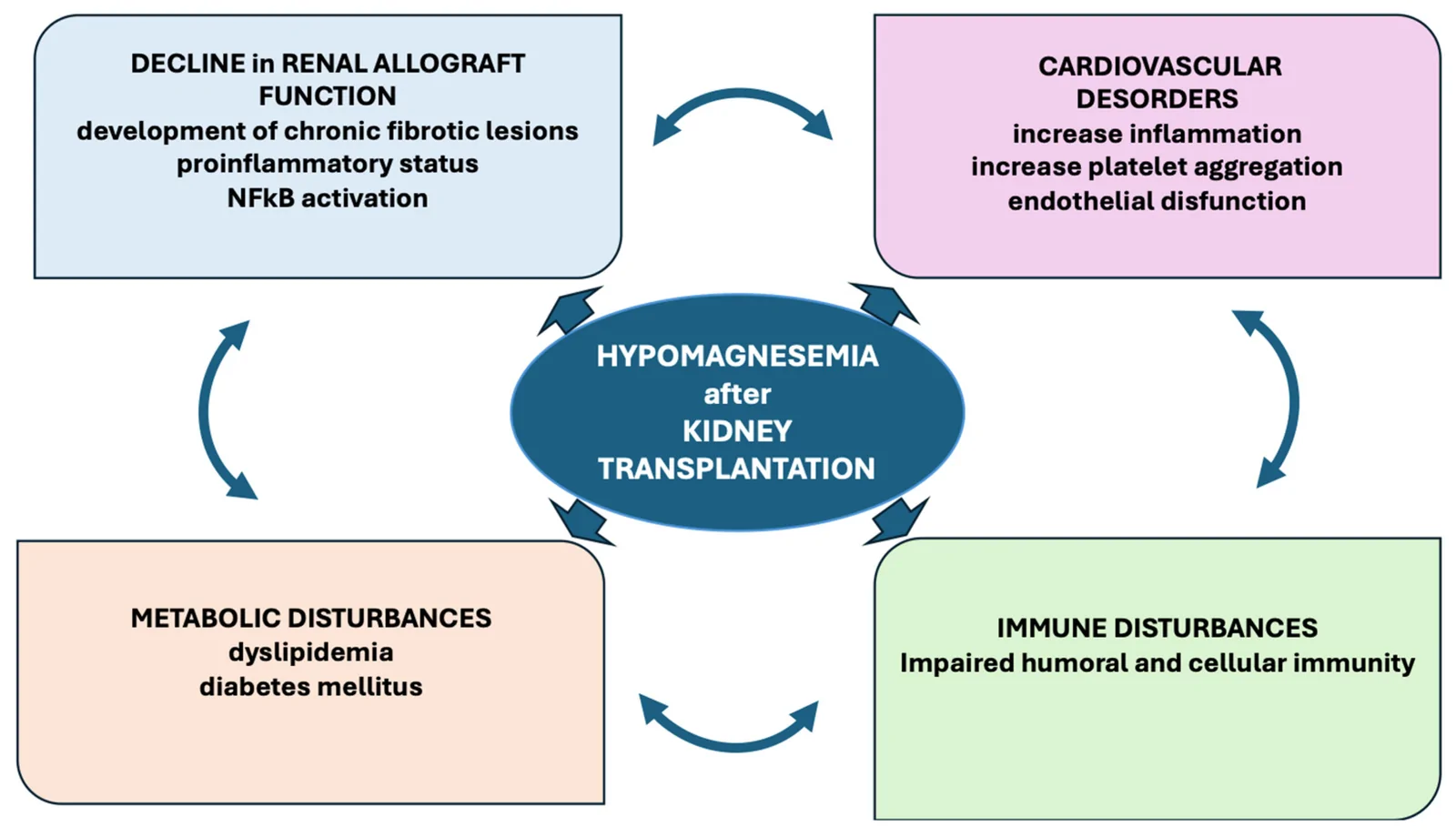
Potential Hypomagnesemia effects after kidney transplantation.

Post-transplant hypomagnesemia has several clinical effects, including neuromuscular hyperexcitability, extremity tremors, weakness, decreased tolerance to physical and mental exertion, insulin resistance, and the development of diabetes mellitus. Additionally, hypomagnesemia may contribute to enhanced oxidative stress and a pro-inflammatory state, with potentially significant cardiovascular implications (Figure 1).

The management of hypomagnesemia, beyond strict monitoring of serum tacrolimus levels, involves correcting the deficit with exogenous magnesium supplementation. Magnesium supplements currently available on the market are highly heterogeneous, ranging from various magnesium salts to magnesium orotate. In vitro bioavailability studies have suggested a superior bioavailability of magnesium orotate; however, the doses required to achieve adequate magnesium repletion may be considerable.

The pharmacological regimen of kidney transplant recipients is typically complex and highly diversified. In addition to the standard triple immunosuppressive therapy—consisting of a calcineurin inhibitor, a purine synthesis inhibitor, and corticosteroids—patients frequently receive antihypertensive agents, gastroprotective and hepatoprotective medications, and lipid-lowering drugs. Consequently, the average number of tablets administered daily often exceeds 15. Furthermore, in more than 20% of patients receiving tacrolimus and mycophenolate mofetil, including formulations with enteric-coated capsules, the administration of oral magnesium preparations is associated with accelerated intestinal transit. Considering these factors, along with the non-negligible financial burden, adding any medication perceived as non-essential to post-transplant outcomes often leads to reduced patient adherence.

The purpose of this study was to develop a topical magnesium-containing lipogel designed to facilitate transdermal magnesium absorption and to provide beneficial effects in alleviating neuromuscular symptoms in kidney transplant recipients with magnesium deficiency, a condition with a well-documented impact on both clinical manifestations and exercise tolerance. In the initial phase, the study evaluates the in vitro bioavailability of the formulation using Franz diffusion cells. In the subsequent phase, the in vivo efficacy of the preparation is assessed in kidney transplant recipients presenting clinically manifest magnesium deficiency that is insufficiently corrected or not correctable through oral magnesium supplementation. Furthermore, the product was designed as a proof-of-concept formulation and a potential alternative to oral magnesium administration, with the present pilot study intended to provide preliminary clinical evidence to guide further development of topical magnesium products. Because the study was not designed to characterize the pharmacokinetic profile or systemic bioavailability of topical magnesium, serial pharmacokinetic sampling, IVPT assessment, and calculation of Cmax, Tmax, t_1/2_, and AUC were not performed; these aspects should be addressed in future dedicated pharmacokinetic and bioavailability studies.

## 2. Materials and Methods

The study was conducted in two phases. The first phase included the development of the topical magnesium-containing formulation and the assessment of its in vitro bioavailability. The second phase consisted of a pilot study in which the lipogel was applied to a selected cohort of kidney transplant recipients presenting with hypomagnesemia resistant to oral magnesium supplementation.

### 2.1. Phase I

#### 2.1.1. Materials

The excipients used in the preparation of lipogels are listed in Table 1. Magnesium orotate was purchased from Merk (Darmstadt, Germany). Magnesium sulphate, Carbomer 940, propylene glycol, triethanolamine, menthol, ethanol, hydrous Eucerin, and purified water were purchased from SC Vitamar IMP.EXP SRL (Bucharest, Romania). Magnesium citrate was acquired from SC Nutramax Trade (Hunedoara, Romania); xanthan gum, olive squalane, *Butyrospermum Parkii* (Shea) Butter and Cosgard^®^ were procured from Ellemental SRL (Oradea, Romania).

#### 2.1.2. Methods

Preparation of lipogels: Three formulations were prepared using Mg compounds, such as sulphate, orotate, and citrate. The composition of the lipogels containing the Mg compounds is presented in Table 1.

Lipogels were realized by way of a hydro dispersion in successive stages, with the objective of forming a homogeneous, stable, and therapeutically effective semi-solid lipogel.

In the first stage, xanthan gum and carbomer were hydrated in approximately 30% of the total amount of water and maintained at room temperature for 24 h, with slow and intermittent manual stirring to achieve proper hydration of the aqueous phase. This step is essential for developing the three-dimensional network required to achieve the gel’s consistency. Further, propylene glycol was added under continuous mixing, followed by the alcoholic menthol solution, both serving as promoters of transdermal permeation, increasing the absorption efficiency of the active ingredients. After complete dispersion, the mixture was neutralized to pH 5.5 by adding a base (triethanolamine). This stabilizes the gelling network and facilitates skin compatibility. In addition, under constant mixing, the lipophilic phase, formed of emollient excipients and penetration promoters, was added. These consist of olive squalane, Eucerin, and shea butter, which help restore the skin’s lipid barrier and the efficient transport of active ingredients. Finally, the antimicrobial agent (Cosgard^®^) was added, followed by dilution with purified water up to the specific final volume. Mg sulphate and orotate were incorporated by direct dissolution into the gel mass. The less soluble Mg orotate was added as a suspension to ensure homogeneous distribution throughout the preparation.

#### 2.1.3. Characterization of Lipogels

Characterization of lipogels was performed according to procedures specified in Chapter 11.8 of the European Pharmacopoeia to assess physical characteristics relevant to the stability and acceptability of the applied formulations [15].

(a) Organoleptic testing

Organoleptic testing represents an essential step in the preliminary evaluation of topical preparations. It provides relevant information regarding the physical aspect, homogeneity, color, and odor of the final product. In the case of lipogels intended for cutaneous application, these characteristics directly influence acceptability, patient compliance, and perceived product quality.

(b) pH determination

Each formulation was assessed for pH using a Hanna Instruments Inc.8417 pH meter (Woonsocket, RI, USA), with the probe immersed directly in the samples after prior calibration.

(c) Colloidal stability of pharmaceutical preparations

The stability of the pharmaceutical preparations was determined by centrifugation using a Hettich Universal 320 R centrifuge (Tuttlingen, Germany). A total of 30 mL of the sample was centrifuged in conical polyethylene tubes at 6000 rpm for 20 min at room temperature (25 ± 1 °C). Time-dependent phase separation was simulated using centrifugation, and the phase stability of the lipogels was evaluated by the stability index (SI). SI was calculated using the following Equation (1):

(1) $$SI\%=\left(1-\frac{V_{2}}{V_{1}}\right)\times 100$$

where *V*_2_ represents the total volume of the lipogel, and *V*_1_ corresponds to the volume of the oil phase (supernatant) after the centrifugation.

(d) Rheological analysis

The rheological analysis, consisting of viscosity and texture profile analyses, is a relevant method for characterizing the resulting lipogels [16].

Viscosity analysis was performed using the RM 200 PLUS Lamy Rheology Instruments (Champagne-au-Mont d`Or, France) rheometer, equipped with an LV-4 spindle. The assessments were performed in triplicate at 5 rpm and 21 ± 2 °C, with results being reported as the mean ± standard deviation (SD).

Texture profile analysis comprised a reproducible evaluation of the mechanical and structural properties of the lipogels. TA.XTPlusC Texture Analyser (Stable Micro Systems Ltd., Godalming, UK) was utilized for the multi-parameter assessment of the lipogels’ texture. Characteristics such as firmness, consistency, adhesiveness, and spreading capacity were studied. A fixed extrusion cell was employed, and the probe was set to penetrate the gel samples within their containers to a depth of 25 mm, at a constant speed of 2.0 mm/s. Force measurements exerted on the probe were recorded via the software Exponent Connect Lite version 8.0.11.0 (Stable Micro Systems Ltd., Godalming, UK).

Spreading capacity testing was performed using a tension fixture equipped with a fixing platform and a conical sample holder. The probe was driven into each gel sample at a constant speed of 2 mm/s, reaching a penetration depth of 10 mm during the measurements. Determinations were carried out in triplicate, with results expressed as mean ± SD. Texture parameters (firmness, consistency, adhesiveness, and spreading capacity) are presented in Table 2.

#### 2.1.4. Determination of In Vitro Release of Mg from Pharmaceutical Preparations

Evaluation of the in vitro release of Mg from topical preparations containing Mg was performed using a six-cell Franz diffusion system with synthetic membranes (Microette-Hanson system, model 57-6AS9, Copley Scientific Ltd., Nottingham, UK) with a surface of diffusion of 1.767 cm^2^ and a volume of 6.5 mL for the receiver chamber [17]. The receptor chamber in each diffusion cell was filled with a phosphate buffer (pH 7.4) mixed with 30% freshly prepared ethanol. Synthetic membranes (Tuffryn^®^, PALL Life Sciences HT-450, lot T72556, Ann Arbor, MI, USA) made of polysulfone with a diameter of 25 mm and a pore size of 0.45 µm were hydrated by immersion in the receptor medium for 30 min before use and then mounted between the donor and acceptor compartments of the Franz diffusion cell. A hydrophilic Tuffryn^®^ polysulfone membrane was selected for the in vitro release study because its primary role was to separate the donor and receptor compartments while providing minimal diffusional resistance. The membrane was not intended to simulate the barrier properties of human skin; therefore, the results represent drug release from the formulation rather than transdermal permeation. Each sample was brought into the diffusion cell capsule at a weight of approximately 0.500 g. The system was maintained at 32 ± 1 °C, and the receptor medium was continuously stirred (600 rpm) using a magnetic stirrer to avoid diffusion layer effects. A 0.5 mL sample of the receptor solution was collected at different intervals of time (30 min, 1 h, 2 h, 3 h, 4 h, 5 h, 8 h, 12 h, and 24 h) to measure the total amount of Mg using the spectrophotometric method. To ensure a consistent volume (6.5 mL) throughout the experiment, a new receptor medium was introduced at the specified intervals instead of using the extracted sample.

#### 2.1.5. Quantification of Mg from Solutions Obtained Following In Vitro Dissolution

A 12 mg/mL Magnesium sulphate stock solution was prepared, from which six aliquot solutions were obtained with the following concentrations: 9.6, 8.0, 6.0, 4.0, 3.0, and 2.4 mg/mL. The calibration curve was determined by reacting Mg ions in the sample with xylenol orange (0.1% solution in an acetate buffer) in a slightly acidic environment, forming a purple complex. The color intensity, proportional to Mg concentration, was measured spectrophotometrically. The absorbance of the solutions was measured at 575 nm using a UV-VIS spectrophotometer equipped with 1 cm quartz cells (UV/VIS Spectrophotometer T70 + PG Instruments Ltd., Wibtoft, UK, Soft UVWIN 5.0, MS Office 2023).

The experimental procedure involved adding 0.5 mL of each Magnesium sulphate solution to 0.5 mL of indicator, then bringing the volume to 3 mL with acetate buffer. The mixture was left to react for 5–10 min at room temperature, after which the absorbance was measured at 575 nm, using a reagent blank as a reference. The obtained calibration curve had a correlation coefficient of 0.99722 over the concentration range of 2.4–9.6 mg/mL. This was used to determine the analogous Mg ions in the analyzed samples [18,19]. All determinations were performed in triplicate.

### 2.2. Phase II: In Vivo Evaluation of the Mg Orotate-Containing Lipogel Effectiveness

We conducted a prospective study on a cohort of 65 kidney transplant recipients with a transplant duration of more than two years, all receiving tacrolimus as the calcineurin inhibitor in their immunosuppressive regimen. The study was conducted in accordance with the Declaration of Helsinki. The therapy satisfaction was conducted following the approval of the Ethics Committee of the Faculty of Medicine and Pharmacy, University of Oradea, under no. 35/30.07.2025 and received approval from the Ethics Committees of the Bihor Country Emergency Clinical Hospital under no. 8039/05.03.2026.

#### 2.2.1. Study Design

**Figure 2:**
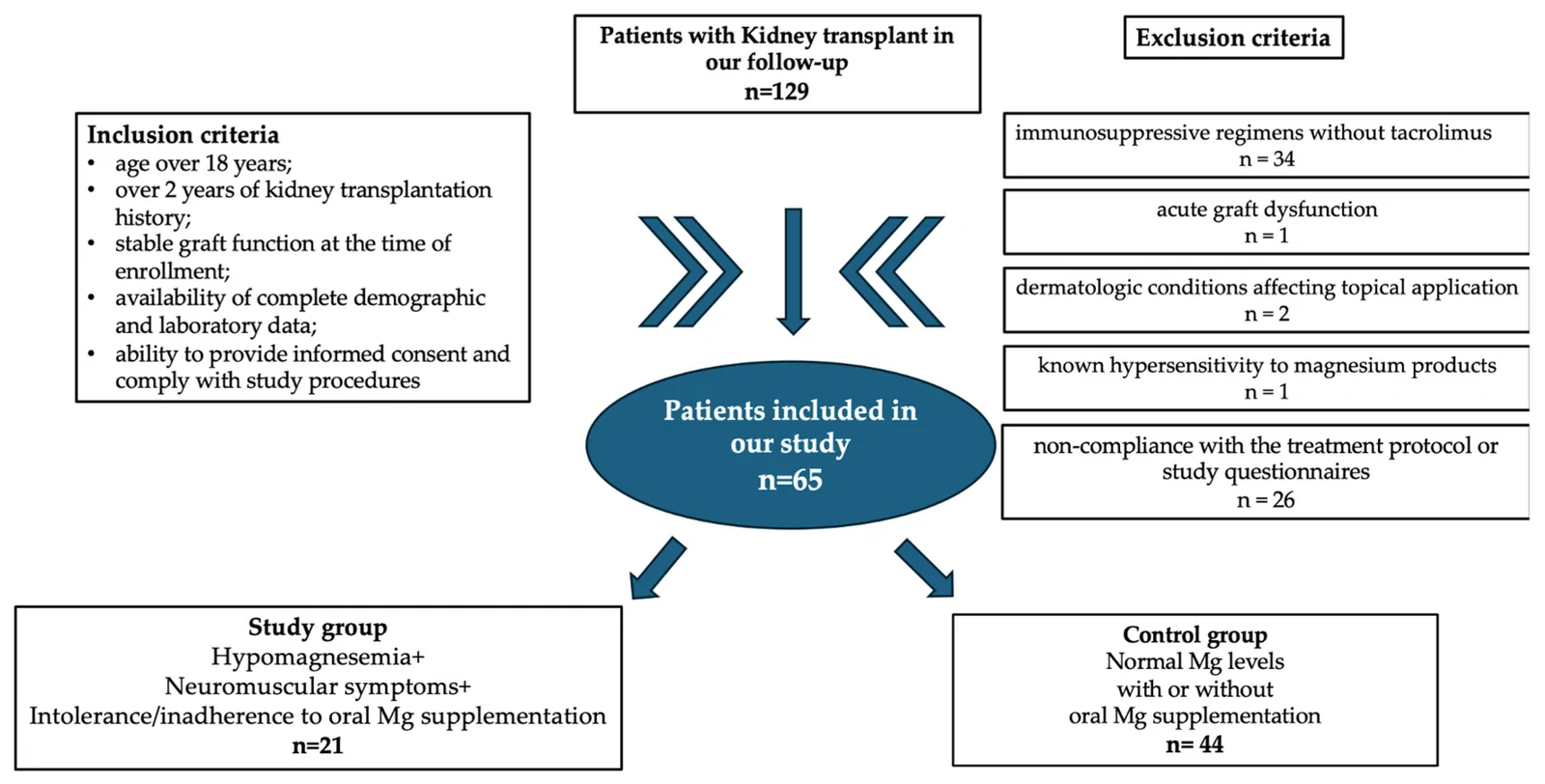
Flow chart with the patient characteristics.

This study was designed as a prospective, controlled pilot study to explore the potential effects of transdermal magnesium orotate supplementation on clinical symptoms and serum magnesium levels and to evaluate its safety profile, particularly with regard to the stability of renal graft function, assessed using standard markers of kidney function during the study period. A total of 65 adult kidney transplant recipients followed at our center were included in the study. Patients were divided into two groups according to their serum magnesium levels. The study group consisted of 21 kidney transplant recipients with documented hypomagnesemia associated with neuromuscular symptoms and intolerance to oral magnesium supplementation. The control group included 44 kidney transplant recipients with normal serum magnesium levels, with or without oral magnesium supplementation (Figure 2).

(a) Inclusion Criteria

- Age ≥ 18 years.
- Over 2 years of kidney transplantation history, with stable graft function at the time of enrollment.
- Availability of complete demographic and laboratory data.
- For the study group: documented hypomagnesemia associated with neuromuscular symptoms and an inability to receive oral magnesium supplementation due to gastrointestinal intolerance and/or financial barriers that limit long-term treatment adherence. For the control group: normal serum magnesium levels, with or without oral magnesium supplementation.
- Ability to provide informed consent and comply with study procedures.

(b) Exclusion Criteria

- Treatment with immunosuppressive regimens without tacrolimus at the time of enrollment.
- Acute graft dysfunction or an acute rejection episode during the study period.
- Severe dermatological conditions affecting the lower limbs that could interfere with the topical application of the study product.
- Known hypersensitivity to magnesium-containing topical formulations.
- Inability to adhere to the topical treatment protocol or to complete study questionnaires.

After the baseline comparison between the two groups, patients in the hypomagnesemia group received a topical magnesium lipogel applied to the calf region twice daily for a period of two months. Patients were instructed to apply an adequate amount of lipogel to cover the entire calf area twice daily, allowing complete absorption without residual oily feeling. The lipogel was applied twice a day to both calves.

#### 2.2.2. Data Collection

(a) Baseline demographics were collected from all participants, including age, sex, time since kidney transplantation, donor type, and employment status. In addition, longitudinal clinical data regarding cardiovascular disease, post-transplant diabetes mellitus, malignancies, and infections requiring hospitalization were documented.
(b) Laboratory parameters were recorded at baseline and after 2 months: serum magnesium, estimated glomerular filtration rate (eGFR), hemoglobin, high-sensitivity C-reactive protein (hs-CRP), total cholesterol, HDL-cholesterol, LDL-cholesterol, triglycerides, total calcium, uric acid, and total serum proteins. Biochemical parameters, including triglycerides, total cholesterol, LDL cholesterol, HDL cholesterol, glucose, creatinine, magnesium, C-reactive protein (CRP), were measured using the Abbott Alinity c analyzer (AC06028) (Abbott Laboratories, Abbott Park, IL, USA) based on the chemiluminescent microparticle immunoassay (CMIA) technique, while hemoglobin levels were assessed using the Abbott Alinity hq analyzer (HQ00687) (Abbott Laboratories, Santa Clara, CA, USA).
(c) To enhance the relevance of the results, we also used the International Physical Activity Questionnaire—Short Form (IPAQ-SF) to assess physical activity levels among the study participants [20]. The questionnaire was administered to two groups of kidney transplant recipients: the study group, consisting of patients with hypomagnesemia, and the control group with normal serum magnesium levels. In this context, several aspects related to the frequency, duration, and intensity of physical activity, as well as the time spent sitting, were evaluated. The questionnaire included items referring to physical activity during the previous seven days and was completed by the patients both before the initiation of treatment with the magnesium lipogel and after the 60-day period during which they applied the preparation. This questionnaire includes information related to the duration, frequency and intensity of intense and moderate physical activities carried out by the patients included in the study. The last question includes information about the time spent sitting. The first question is related to the number of days in which the patients carried out intense physical activity (lifting weights, fast running, cycling, aerobics, various performance sports) in the last 7 days. The second question refers to the time (minutes) in which they carried out these intense activities in the 7 days. The third question refers to the number of days with moderate activities (lifting weights under 10 kg, light exercises, cleaning the house) in the last 7 days. The fourth question refers to the time (minutes) in which they carried out moderate physical activities in the last 7 days. The fifth question refers to the number of days in which they walked in the last 7 days, and the sixth question includes the number of minutes of walking in the last 7 days. Question number 7 refers to the time spent sitting (minutes) at home or at work, but not including sleep, on one day in the last 7 days.

We calculated MET (Metabolic Energy Turnover) expressed as MET-minutes per week. For this purpose, moderate physical activity was assigned a value of 4 METs. To determine the weekly MET-minutes, the assigned MET value (intense activity = 8, moderate activity = 4, walking = 3.3) was multiplied by the duration of the activity in minutes and by the number of days per week during which the activity was performed. By summing the resulting MET-minutes, we obtained for each patient the total weekly MET-minutes of physical activity. For the purposes of classification, we considered the following criteria: vigorous physical activity performed for at least 20 min per day on at least 3 days per week, moderate physical activity performed for at least 30 min per day on at least 5 days per week, or a combined physical activity level reaching at least 600 MET-minutes per week over a minimum of 5 days [21].

(d) Patient satisfaction with therapy

To assess patients’ satisfaction with the magnesium orotate-containing lipogel, we used the LMSQ, an internationally validated questionnaire (Table S1, Supplementary Materials). The questionnaire consists of 18 statements (LMSQ_1 to LMSQ_18), each rated on a four-point Likert scale from one to four. 1 = Strongly disagree, 2 = Disagree, 3 = Agree, 4 = Strongly agree. Statements LMSQ_2, LMSQ_9 and LMSQ_17 are marked with an asterisk, indicating that they have been negatively worded and therefore should be reversed before rating. Prior to completing the questionnaire, all 21 participants were informed about the purpose of the study, the voluntary nature of their participation, and the confidentiality of their responses, and they provided written informed consent. Throughout the study, subjects maintained the same lifestyle and did not use other topical preparations for at least one month before the study or during the study (Table S1).

We selected the clinical outcomes based on their relevance to the main objectives of the study: assessing the acceptability of the topical magnesium orotate formulation and exploring its potential impact on symptoms associated with magnesium deficiency, particularly neuromuscular symptoms and physical performance. The selected outcomes were therefore intended to capture patient-relevant functional changes rather than transplant-specific endpoints. These parameters were chosen to provide preliminary clinical evidence to support further evaluation of this topical formulation.

#### 2.2.3. Statistical Analysis

The statistical analysis was performed using JAMOVI software version 2.7.2. Statistical significance was set at a two-sided *p*-value < 0.05. Continuous variables were presented as mean ± standard deviation (SD), and categorical variables were reported as frequencies (counts) and percentages. The distribution of continuous variables was assessed using the Shapiro–Wilk test. Non-normally distributed variables were reported as the median and interquartile range. For comparisons between two independent groups, the independent-samples *t*-test was used for normally distributed variables, while the Mann–Whitney U test was applied to non-normally distributed variables. In interpreting the findings, statistical significance was considered together with the magnitude and variability of the observed differences. To evaluate whether the LMSQ questionnaire was suitable for the purpose of the study and the patient sample, the Kaiser–Meyer–Olkin (KMO) measure of sampling adequacy and Bartlett’s test of sphericity were performed. To compare scores across respondent categories in the study, the *t*-test or ANOVA with Bonferroni post hoc tests was used. Categorical variables were presented as counts (percentages) and compared using chi-square tests.

## 3. Results

### 3.1. Phase I

#### 3.1.1. Organoleptic Testing

*Aspect and homogeneity*: The lipogels presented a homogeneous appearance, without visible particles or separate phases, reflecting a uniform dispersion of excipients and active ingredients, indicating a correctly formulated formulation. The color of a topical product is required to be consistent, uniform, and consistent with its composition. In the case of Mg formulations, pearly white or opaque white was typical of inorganic salts and pigment-free lipogels. Color changes could signal ingredient degradation or contamination. The odor was required to be pleasant, specific to the formulation, without rancid or fermented notes, which could indicate microbiological alterations or oxidation of the fatty phase. The presence of menthol conferred a characteristic, cosmetically acceptable odor, contributing to the sensation of freshness and comfort upon application. A rigorous organoleptic control allowed early identification of non-conformities in the manufacturing process, ensuring visual and sensory stability during the shelf life, increasing user confidence in the product and compliance with treatment. Thus, organoleptic evaluation, although considered a routine control, played an essential role in verifying the overall quality of lipogel-type dermato-cosmetic and pharmaceutical products. The main organoleptic characteristics of the three lipogel formulations are presented in Table 3.

#### 3.1.2. pH Determination

The pH of a topical preparation is a critical parameter in cosmetic dermatology formulation, influencing both cutaneous tolerability and the stability, as well as efficacy of active ingredients. In the case of Mg lipogels, maintaining an optimal pH was essential to ensure compatibility with the physiological skin barrier and to avoid irritation or functional alterations. The human skin has a naturally slightly acidic pH, with values between 4.7 and 5.75, known as the “acid mantle”. This physiological acidity maintains the integrity of the hydrolipidic film, inhibiting the proliferation of pathogenic microorganisms and the optimal functioning of epidermal enzymes involved in keratinocyte differentiation and corneocyte formation.

In the analyzed formulations, the lipogels presented a pH of 5.5, a value considered appropriate for cutaneous application, ensuring efficient absorption of Mg salts (sulfate, citrate, orotate) and increased tolerability, without affecting the acid balance of the skin and concomitantly minimizing the risk of irritation, especially in case of repeated use.

A more alkaline pH (>6) could compromise the epidermal barrier, while a markedly acidic pH (<4.5) could cause burning or irritation sensations, especially on sensitive or damaged skin. Therefore, the choice of a pH of 5.5 for Mg lipogels was justified from a pharmaceutical and dermatological point of view, maintaining the balance between therapeutic efficacy and cutaneous safety.

#### 3.1.3. Colloidal Stability of the Pharmaceutical Preparations

The diverse composition of the preparation alongside the obtaining procedure can influence the colloidal stability, the ratio between the aqueous and lipophilic phases, causing changes in the stability of the preparation.

All the planned formulations showed high stability; the stability index (SI) was determined to be 100%. A crucial role in the stability of lipogels could be attributed to the emulsifier and thickening agent. Eucerin is a lipophilic excipient with emulsifying properties that stabilizes the oil–water interface and supports the consistency of the formulation. Carbomer is a synthetic polymer of polyacrylic acid with high efficacy as a gelling agent, providing pharmaceutical preparations with high viscosity at low concentrations.

#### 3.1.4. Rheological Characterization of the Lipogels

The physical characteristics of lipogels had a particularly marked impact on the stability, properties, and acceptability of topical preparations. This led us to quantify them using texture analysis using probes that penetrate the sample. Thus, values for force, distance, and time were recorded to assess the quality of the developed samples. It was also important to correlate the sensory and textural properties analyzed so that the topical products could be accepted by the study subject upon use. Table 4 presents the mean values of triplicate determinations ± standard deviation (SD) for the three lipogel formulas obtained.

Based on the results, the lipogels exhibited similar textural properties, including viscosity, consistency, and firmness. The adhesiveness and spreading capacity differed among the three formulations due to the type of Mg salt incorporated, confirming the importance of selecting the appropriate salt in a topical formulation. Therefore, the adhesiveness values were lower, while applicability was less influenced. The three formulas had similar values for this parameter, without significantly affecting the rheological behavior.

#### 3.1.5. Quantification of Mg from Solutions

**Figure 3:**
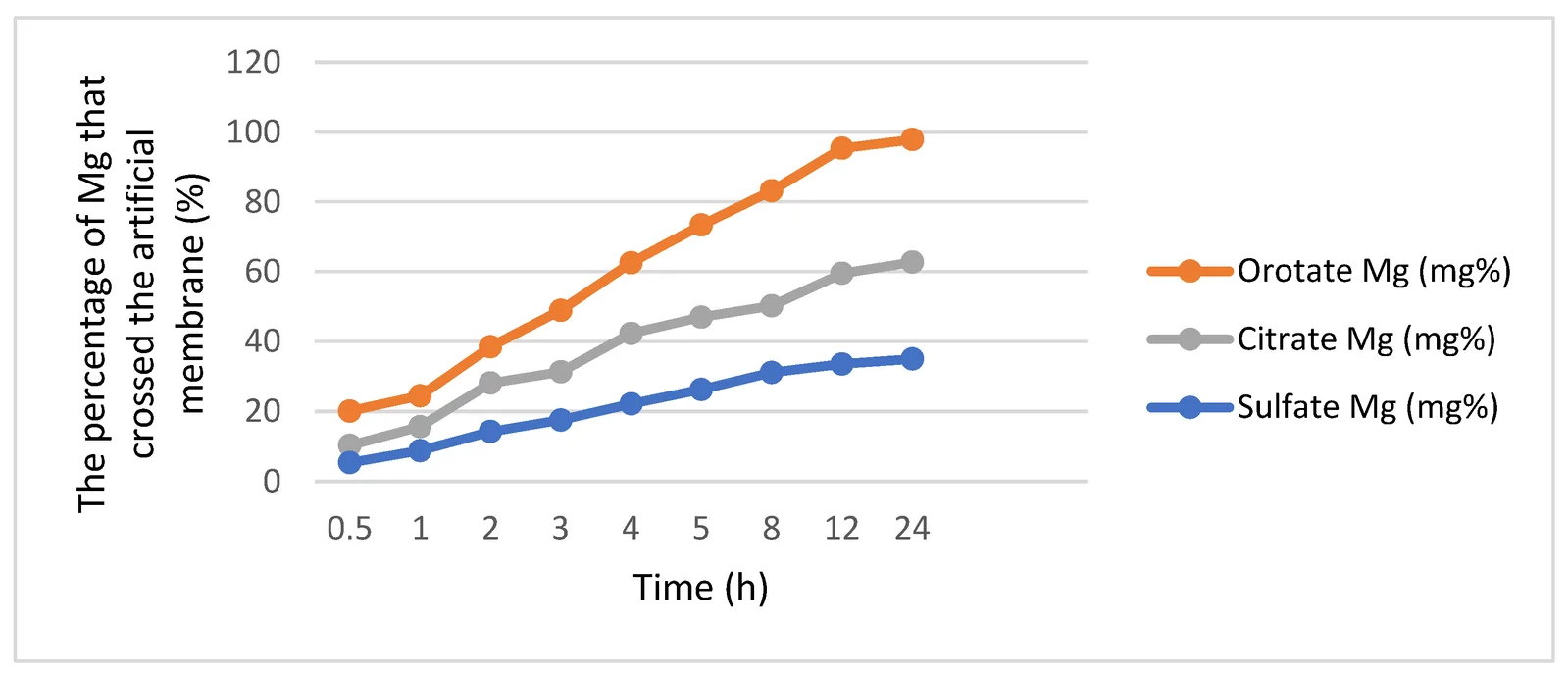
Magnesium (Mg) Permeation Across the Artificial Membrane. Results are presented as mean ± standard deviation (SD). Statistical analysis was performed using one-way ANOVA, followed by Tukey’s multiple comparison test. Statistical significance was set at *p* < 0.05.

The therapeutic efficacy of the lipogel is directly proportional to the amount of Mg that crosses the epithelial barrier. In this sense, the amount of Mg in the acceptor solution following diffusion through the membrane at different time intervals was determined. All determinations were performed in triplicate. The results are displayed in Figure 3.

The release of the active ingredient from the formulation and its permeation through the skin are paramount as they characterize the preparation’s therapeutic efficacy. Given that the excipients used in the formulations are identical and in the same quantity, the differences in the ability of Mg to penetrate the skin are due exclusively to the Mg compound in each formulation.

As shown in Table 5, there are notable differences among the three formulations in terms of the penetration rate of Mg through the artificial membrane. These differences are maintained throughout the determination period, and results after 24 h show that the highest penetration rate of Mg was observed for Mg orotate (97.84%), while the lowest was for Mg sulfate (35.05%). Taking these differences into account, the decision was made to conduct the clinical study only with the preparation containing Mg orotate as the active ingredient, since penetration rates of 35% and 63% were considered insufficient to achieve a therapeutic effect and conclusive results. The use of a Tuffryn^®^ membrane allowed for the evaluation of the formulation’s release characteristics; however, it does not replicate the barrier properties of human skin. Therefore, we are considering future additional studies using skin-mimicking membranes—such as Strat-M^®^ or excised skin—to evaluate transdermal permeation.

### 3.2. Phase II Clinical Study

#### 3.2.1. Characteristics of the Study Cohort

The study cohort had a mean age of 50.95 ± 10.16 years and was predominantly male (66.7%). Most patients (80.95%) received a kidney graft from a deceased brain-dead donor. Only one-third were employed, with the remainder being retirees. Post-transplant diabetes mellitus occurred in 33.3% of patients, a prevalence comparable to that of severe infections requiring hospitalization. Neoplasms were identified and treated in two patients, while 23.8% had cardiovascular conditions requiring ongoing monitoring and management. All patients demonstrated stable renal function, with a mean eGFR of 60.72 ± 24.95 mL/min/1.73 m^2^, serum magnesium 1.45 ± 0.11 mmol/L, hemoglobin 13.54 ± 1.72 g/dL, and CRP 5.61 ± 5.26 mg/L (Table 6).

#### 3.2.2. Laboratory Parameters After Topical Usage of the Mg Orotate-Containing Lipogel

At 60 days following the administration of the lipogel containing magnesium orotate, serum magnesium, creatinine, estimated glomerular filtration rate (eGFR), high-sensitivity C-reactive protein (hsCRP), and hemoglobin levels were evaluated. Because the distributions of magnesium and hemoglobin values did not meet the normality assumption, non-parametric statistical methods were used. Comparisons were performed using the Wilcoxon test, and the results were expressed as the median and interquartile range (IQR) (Table 7).

Serum magnesium levels increased significantly compared to baseline; however, values remained below the normal reference range (median/IQR 1.47/0.18 vs. 1.55/0.15, *p* < 0.001).

**Figure 4:**
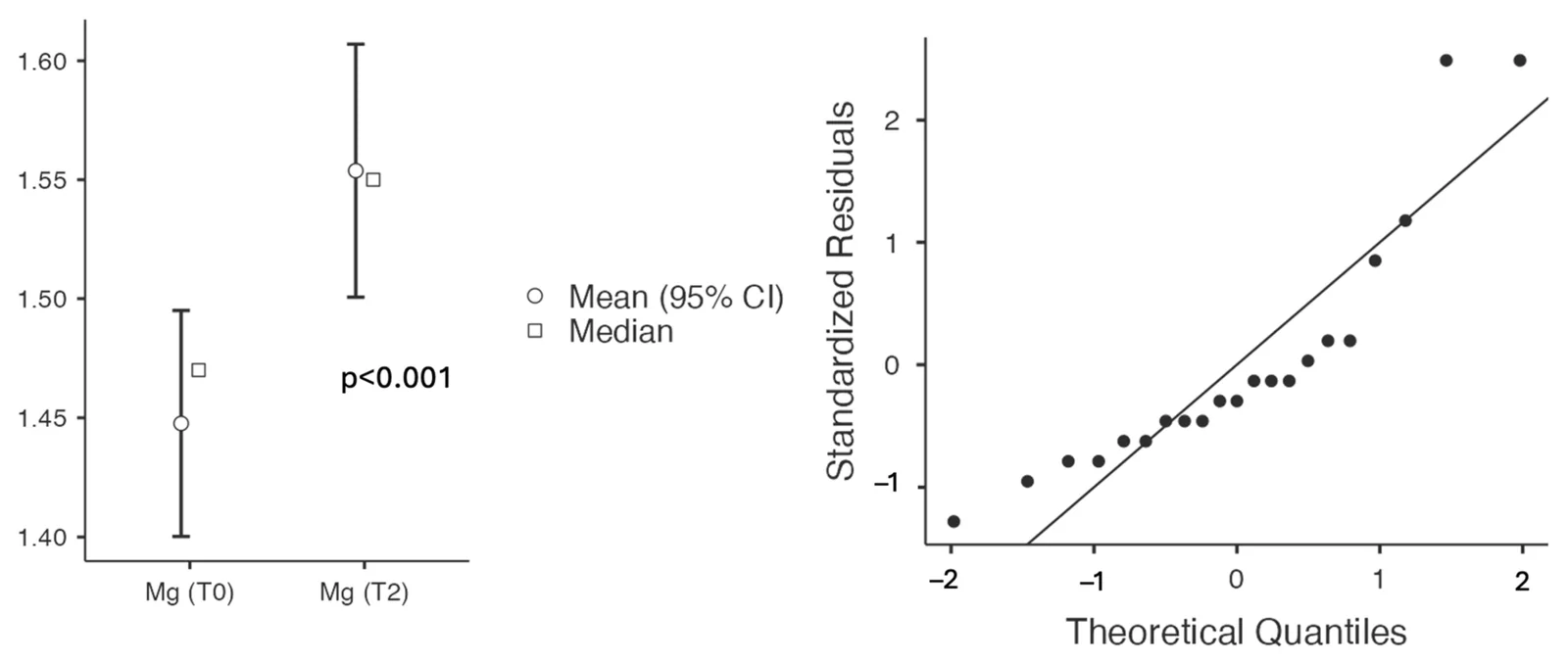
Magnesium value before and after the topical use of Magnesium orotate lipogel.

**Figure 5:**
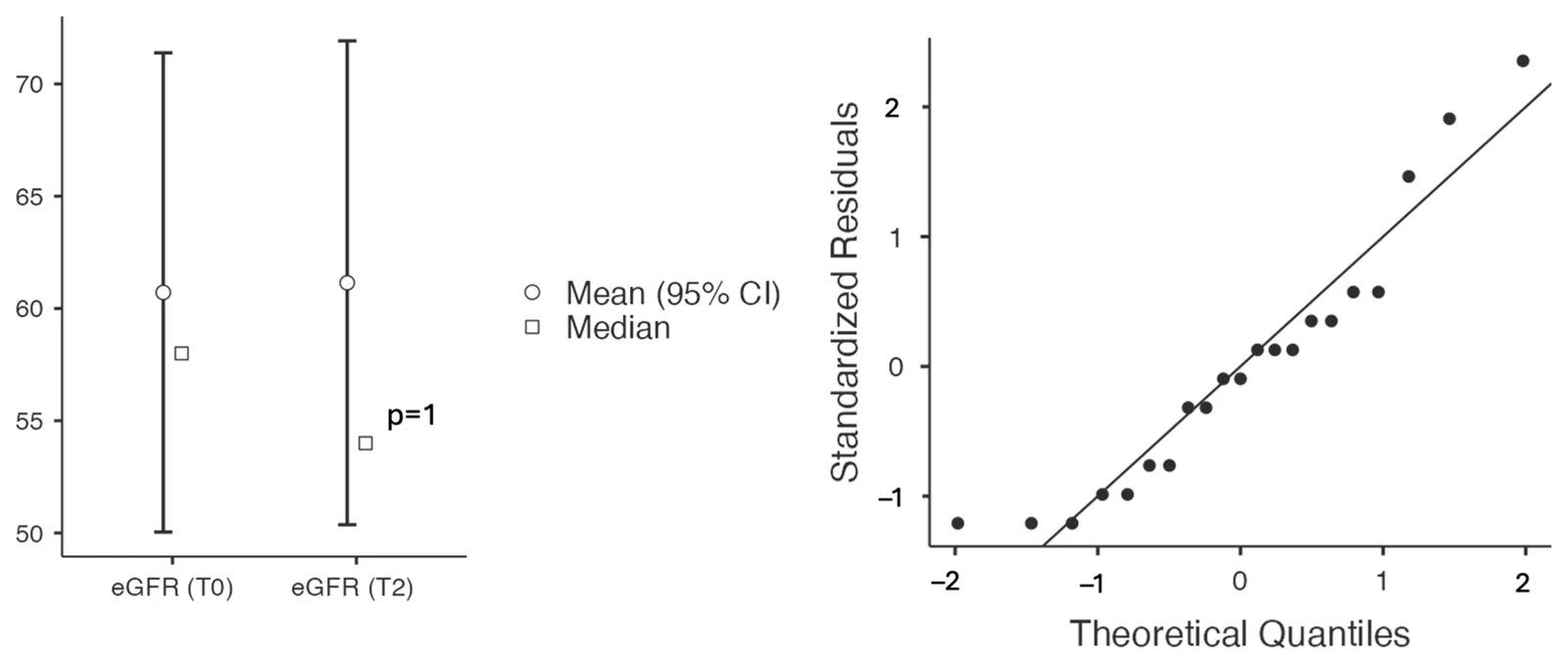
eGFR value before and after the topical use of Magnesium orotate lipogel.

**Figure 6:**
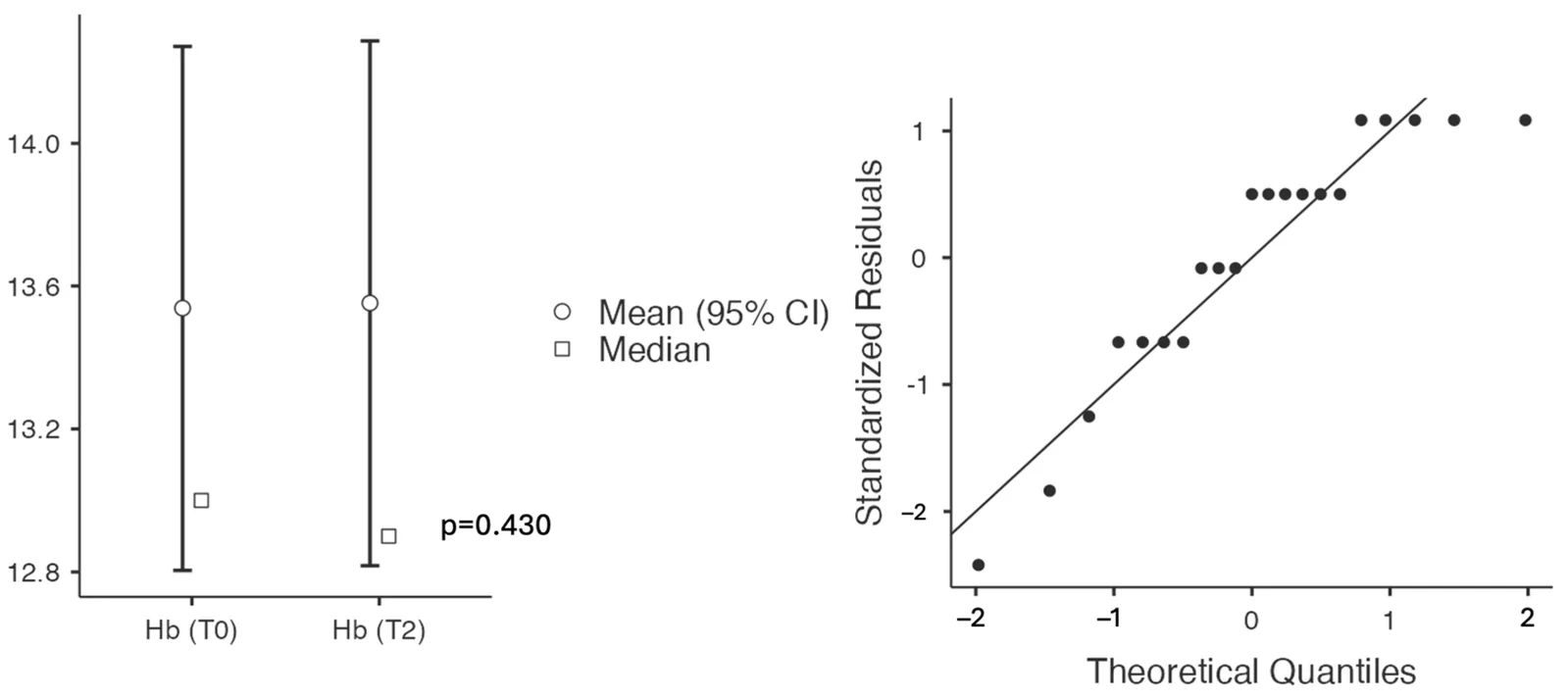
Hemoglobin value before and after the topical use of Magnesium orotate lipogel.

**Figure 7:**
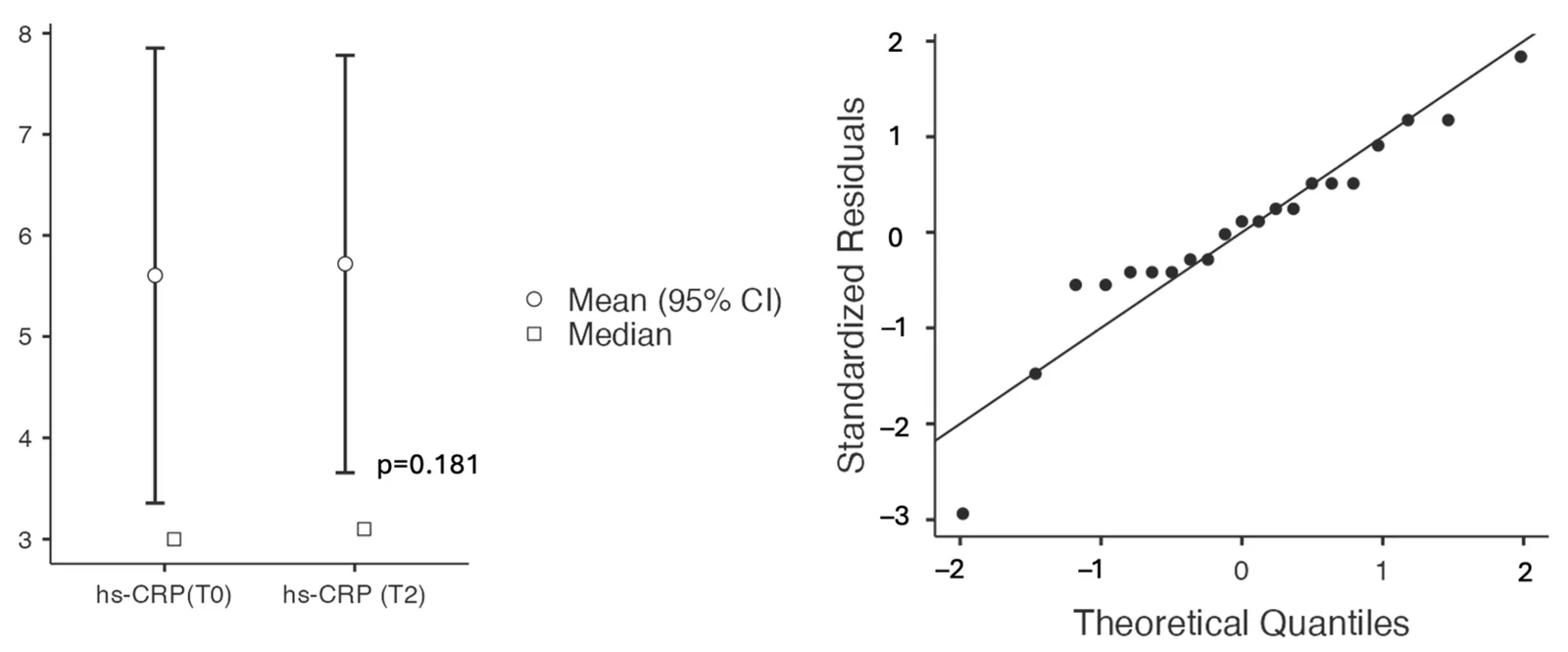
hs-CRP value before and after the topical use of Magnesium orotate lipogel.

Creatinine levels showed a small but statistically significant difference (1.404 ± 0.478 vs. 1.420 ± 0.496, *p* = 0.040). No statistically significant differences were observed for eGFR (61.143 ± 25.19 vs. 60.715 ± 24.948, *p* = 1.000), hsCRP (5.719 ± 4.824 vs. 5.605 ± 5.258, *p* = 0.181), or hemoglobin (median/IQR, 13/2.3 vs. 12.9/2.1, *p* = 0.430) (Figure 4, Figure 5, Figure 6 and Figure 7).

#### 3.2.3. Functional Exercise Capacity Assessment

##### Exercise Tolerance in the Study Group Before and After Magnesium Lipogel Application

Following daily administration of the lipogel containing magnesium orotate, questionnaire-based assessment of exercise capacity demonstrated a significant increase in moderate-intensity physical activity (IPAQ4 310.475 ± 92.505 vs. 279.286 ± 89.907, *p* < 0.001), with no change in vigorous-intensity activity. Time spent in a seated position decreased significantly (256.429 ± 80.330 vs. 284.048 ± 87.029, *p* < 0.001), while total weekly physical activity (MET-min/week) increased significantly following the intervention (7766.286 ± 2920.815 vs. 6764.9 ± 2719.09, *p* < 0.001) (Table 8).

##### Exercise Tolerance in the Study Group Compared with a Control Group

(a) To validate the IPAQ-derived results, a control group of renal transplant patients with serum magnesium levels within the normal range was selected, and the same questionnaires were administered. The values obtained in the control group were used as reference benchmarks for assessing exercise capacity in the study group (Table 9).

The control group did not differ significantly from the study group. Patient age (50.068 ± 12.422 vs. 50.952 ± 10.161, *p* = 0.778), sex distribution (65.81% vs. 66.66%, *p* = 0.448), transplant duration (13.023 ± 4.791 vs. 13.714 ± 5.640, *p* = 0.623), and donor type (75% vs. 76.19%, *p* = 0.917) were comparable between groups. Laboratory parameters were also similar, except for serum magnesium levels (1.791 ± 0.145 vs. 1.448 ± 0.111, *p* < 0.001, which differed significantly. Hemoglobin levels (13.177 ± 1.617 vs. 13.538 ± 1.715, *p* = 0.412), CRP (4.691 ± 3.790 vs. 5.605 ± 5.258, *p* = 0.715), total protein (7.04 ± 094 vs. 6.890 ± 0.348, *p* = 0.221), total calcium (9.245 ± 0.672 vs. 9.190 ± 0.551, *p* = 0.748), and lipid profile were comparable between the two groups. No significant differences were observed in the incidence of comorbidities, including diabetes mellitus (13.63% vs. 33.33%, *p* = 0.063), cardiovascular disease (6.18% vs. 23.8%, *p* = 0.051), neoplasms (6.81% vs. 9.52%, *p* = 0.655), or infections requiring hospitalization (22.72% vs. 33.33%, *p* = 0.363).

**Figure 8:**
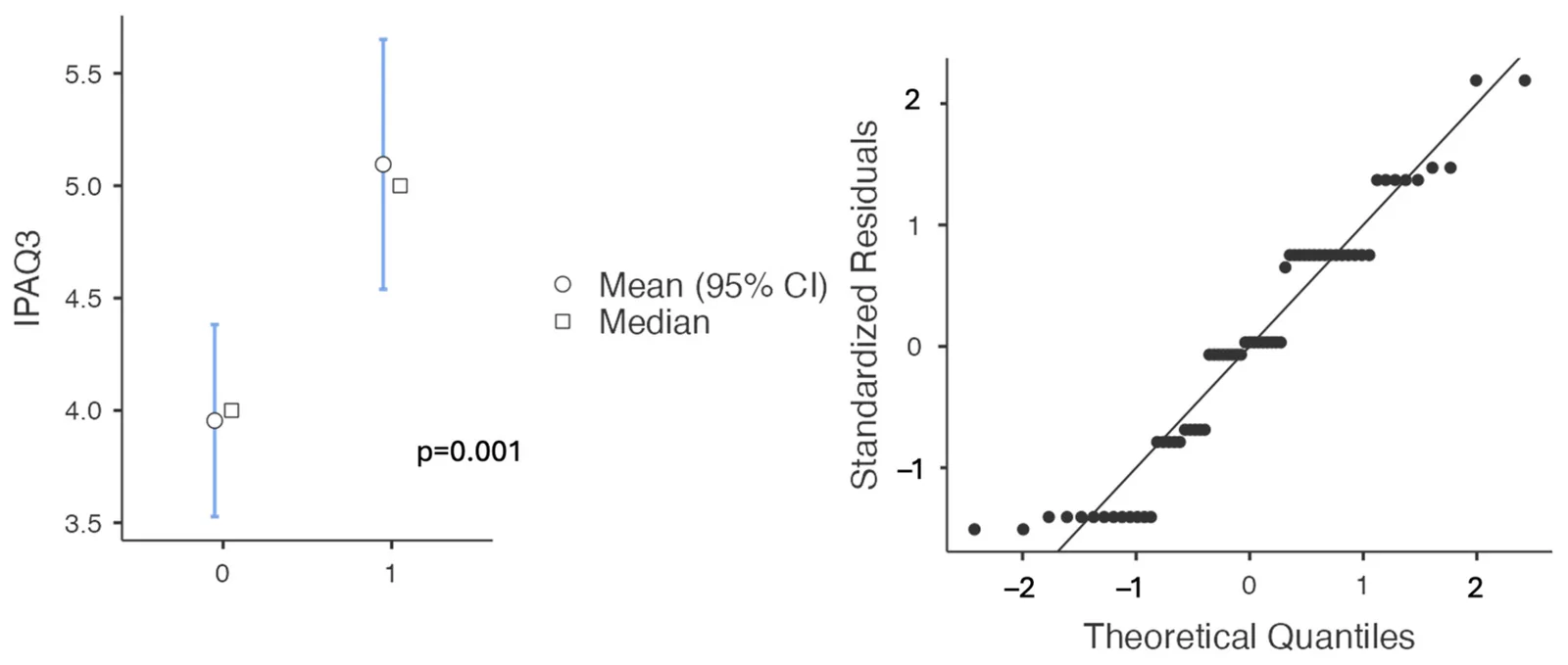
IPAQ3 value before and after the topical use of Magnesium orotate lipogel.

**Figure 9:**
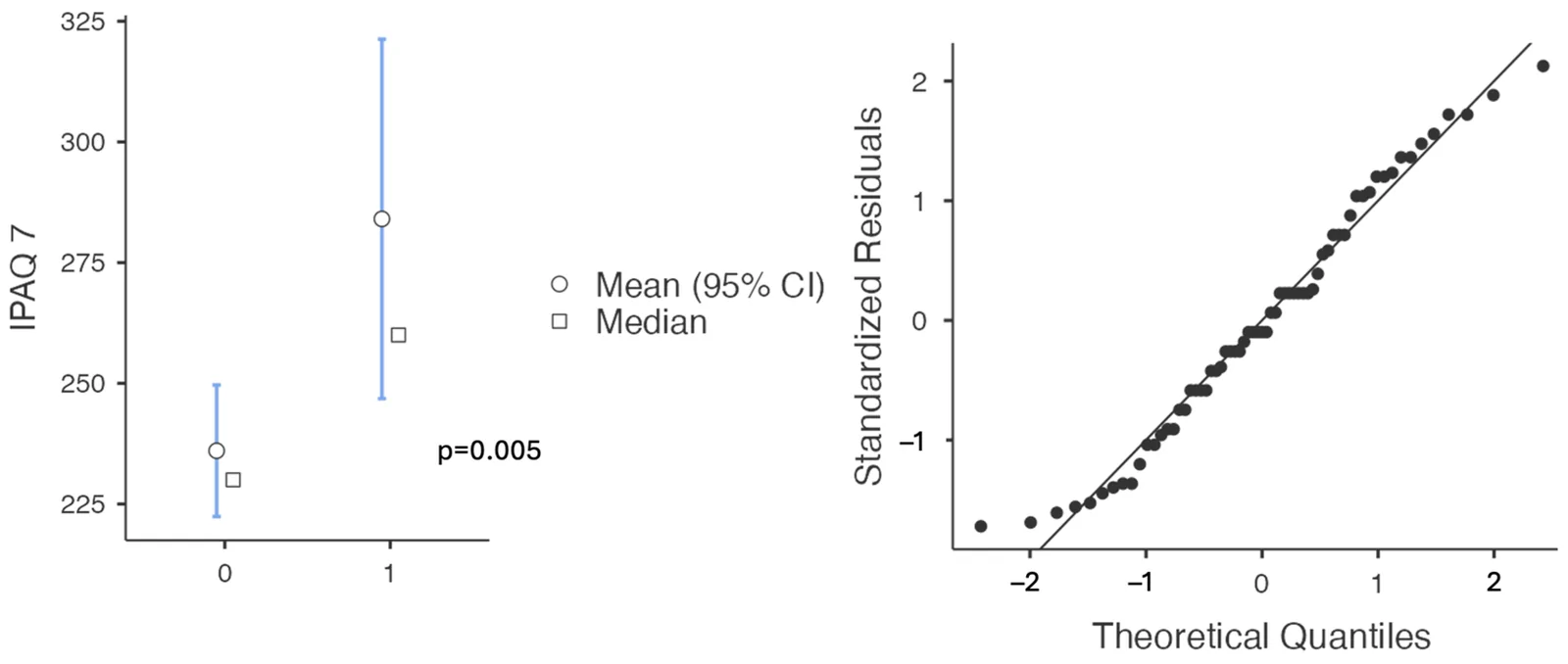
IPAQ7 value before and after the topical use of Magnesium orotate lipogel.

**Figure 10:**
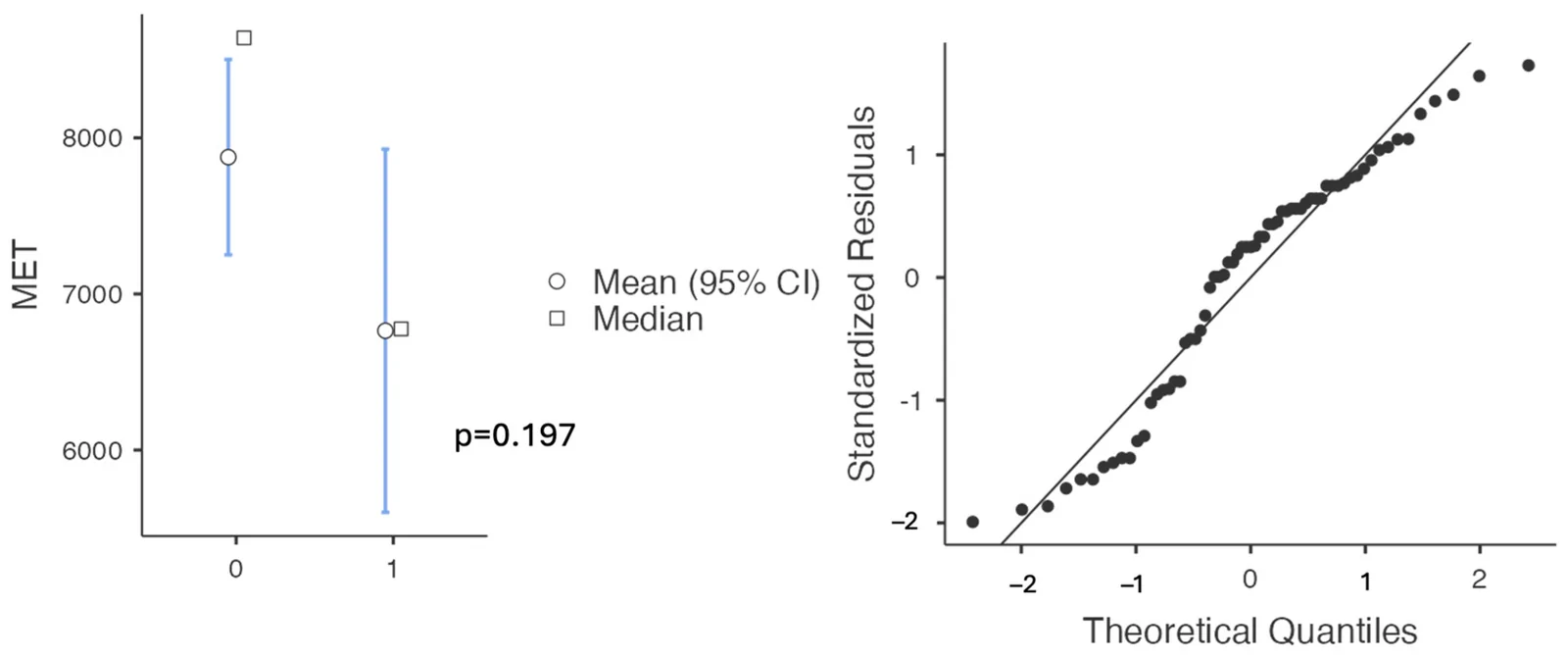
MET value before and after the topical use of Magnesium orotate lipogel.

(b) Although total physical activity, expressed as MET-min/week, was comparable between groups (7766.286 ± 2920.815 vs. 7875.909 ± 2177.947, *p* = 0.822), significant differences were observed in specific activity domains, indicating differences in the pattern of physical activity rather than in its overall volume. Compared with the control group, patients with hypomagnesemia exhibited lower tolerance to moderate-intensity physical activity than those with normal serum magnesium levels (5.190 ± 0.873 vs. 5.818 ± 0.390, *p* = 0.001). In addition, they spent more time seated than patients with normal magnesium levels (284.048 ± 87.029 vs. 236.03 ± 46.086, *p* = 0.005). (Table 10 and Figure 8, Figure 9 and Figure 10).

Following administration of the magnesium-based lipogel, scores improved with respect to moderate-intensity physical activity (IPAQ4 310.475 ± 92.505 vs. 298.977 ± 71.150, *p* = 0.574), and time spent in a seated position decreased (256.429 ± 80.330 vs. 236.03 ± 46.086, *p* = 0.198). Although total physical activity quantified in MET-minutes increased, the change did not reach statistical significance compared with the normomagnesemic control group (7766.286 ± 2920.815 vs. 7875.909 ± 2177.947, *p* = 0.822) (Table 11).

##### Assessment of Factors Influencing the Improvement in MET Following Administration of the Magnesium-Based Lipogel

A multiple linear regression analysis was performed to evaluate whether changes in serum magnesium (ΔMg) and renal function (ΔeGFR) predict changes in exercise capacity (ΔMET) in 21 patients. The overall model was statistically significant (F (2,18) = 22.35, *p* < 0.001), explaining a substantial proportion of the variance in ΔMET (R^2^ = 0.713). Within the model, ΔMg was a strong and statistically significant predictor of ΔMET (β = 2920.43, SE = 438.82, 95% CI 1998.50 to 3842.35, *p* < 0.001), with a large, standardized effect size (β = 0.865). In contrast, ΔeGFR was not significantly associated with ΔMET (β = −7.32, SE = 7.58, 95% CI −23.24 to 8.61, *p* = 0.347). Model assumptions were met, with no evidence of multicollinearity (VIF = 1.06) and normally distributed residuals (Shapiro–Wilk *p* = 0.233) (Table 12).

##### Measurement of Therapy Satisfaction and Physical Activity Tolerance

Subjective evaluation of topical magnesium orotate showed no notable adverse effects. Efficacy, assessed by improvements in muscular fatigue, was rated highly (3.841 ± 0.227), approaching the maximum score, with no significant differences observed according to sex (3.667 ± 0.346 vs. 3.905 ± 0.252, *p* = 0.132) or employment status (3.905 ± 0.252 vs. 3.667 ± 0.346, *p* = 0.132). Practicality of use was high (3.873 ± 0.166), with slightly higher scores reported by male participants (3.929 ± 0.142 vs. 3.762 ± 0.163, *p* = 0.033). The use of this form of magnesium did not substantially interfere with daily activities. Overall satisfaction scores were high (3.894 ± 0.205), with no significant differences by sex (3.877 ± 0.111 vs. 3.929 ± 0.095, *p* = 0.524) or employment status (3.937 ± 0.081 vs. 3.873 ± 0.114, *p* = 0.226). Regarding treatment tolerability and patient satisfaction, all participants (21/21; 100%) reported no adverse effects during the treatment period and therefore assigned the maximum score of 4 for the “Side effects” item. The “Healthcare” and “General satisfaction” items also received the maximum score of 4 from all participants. (Table 13).

## 4. Discussion

One of the important functions of the skin is to act as a barrier, limiting the passage of various substances. To obtain a pharmaceutical preparation with appropriate quality and efficacy, it is necessary to achieve an optimal composition in terms of emulsifier and excipient selection, while also avoiding interactions among formulation components. The pH of the skin ranges from 4 to 5.5 at maturity and is essential for the development of a microbiota (*Staphylococcus epidermidis* and *Corynebacteria*), which helps prevent the growth of fungi and pathogenic bacteria at this level. Frequent use of cosmetic preparations that imprint alkaline pH on the skin leads to multiplying and subsequently penetrating pathogenic bacteria inside the body [22,23].

The excipients used to obtain the topical preparations allowed the maintenance of the semi-solid form, spreading and adhesiveness on the surface of the skin. These excipients formed a three-dimensional, lax network, in which the solutions of Mg citrate and sulfate were found, which formed Van der Waals and hydrogen bonds, obtaining a stable H/L emulsion. Magnesium orotate was suspended in lipogel. Lipophilic excipients give a pleasant appearance to the skin, improving skin elasticity.

The administration of various compounds in semisolid formulations is frequently used in dermatological therapy, although clinical outcomes depend on many factors. These include the physicochemical properties of the active ingredient (solubility, molecular weight, lipophilicity), the properties of the excipients involved in release, and active ingredient permeability. Another equally critical aspect concerns the skin barrier function and the possible changes that result from acute or chronic pathologies. To achieve an effective and appropriate pharmaceutical preparation, it is also important to consider the rheological properties, homogeneity, pH, the absence of interactions among the mixture’s ingredients, the particle size of the active ingredient, and the stability of the final product [24,25].

Emulgels that have an external gelled phase with gelling agents offer control over the release of the active ingredient due to this gel structure responsible for the stability of the emulsion [26]. The presence of water in the gel’s composition favored not only the solubilization of Mg citrate and sulphate but also an adequate hydration of the skin. Due to the small particle size of the active ingredients incorporated in solution form, their passage through the skin is easy and generates an immediate therapeutic effect [27]. Emulgels are a combination of emulsions and gels and offer advantages related to thixotropy, good skin spreadability, increased stability, and high patient acceptability [28]. The composition of the formulated pharmaceutical products ensured both the Mg-specific therapeutic effect and an emollient, skin-hydrating action, due to the selected excipients. This finding is clinically relevant as dehydrated, less elastic skin is not merely a cosmetic concern, but may reflect impairment of the stratum corneum, with a consequent reduction in its barrier function [29]. Whereas excipients primarily provide emollient and moisturizing effects at the stratum corneum, the active substances must traverse this barrier and reach deeper cutaneous layers, including the cellular level, to elicit their therapeutic effect. The extent of penetration is influenced by the physicochemical properties of the active ingredients as well as by the integrity and physiological condition of the skin [30].

Eucerin binds to water-soluble Mg salts and promotes their absorption through the skin. Furthermore, the composition is similar to that of the stratum corneum of the skin, thereby permitting hydrophilic compounds in the pharmaceutical preparation to cross this layer. Eucerin exhibits an occlusive action that reduces transepidermal water loss, thereby helping maintain skin moisture [31].

Propylene glycol in the formulation proposed in this study acts as a dispersing agent and as a hydrophilic absorption promoter by dissolving keratin and interacting with the polar groups of lipids at the intercellular level, thus reducing the barrier function of the stratum corneum [32].

The incorporation of carbomer into the lipogel formulation confers bioadhesive properties and enhances thermal stability. Its broad compatibility with numerous active ingredients, together with its potential for low cutaneous irritation, has contributed to its widespread use in a variety of pharmaceutical formulations. In the proposed formulation, carbomer enhances the physicochemical stability of the preparation by increasing the lipogel’s viscosity, thereby prolonging residence time at the application site. As a result, the bioavailability of the active ingredient may be improved [33]. Maintaining stability is a very important aspect and various excipients are used for this purpose [34].

Xanthan gum is a natural polysaccharide, non-toxic, biodegradable, and water-soluble, displaying pseudoplastic behavior even at low concentrations, and is stable at any pH and temperature. These characteristics stabilize the lipogel and also lead to the controlled release of the active ingredient from the composition [35].

Olive squalane has an emollient and moisturizing effect on the skin. For this reason, it is frequently used in the formulation of lipid emulsions from which it is quickly absorbed into the deep layers of the skin, helping to restore its flexibility and suppleness [36]. Shea butter is an emollient and moisturizer for the skin, but also has antibacterial and antifungal effects [37].

The effectiveness of Mg supplementation is well known and has been proven by numerous scientific studies. To achieve an optimal serum Mg level, various compounds are used. Nevertheless, there are significant variations in serum Mg levels resulting from supplementation with different pharmaceutical forms. Unlike oral Mg administration, transdermal absorption lacks the side effects specific to the gastrointestinal tract.

The differences in Mg bioavailability following administration as different compounds have been studied by many researchers. The results confirm that Mg citrate and orotate are organic formulations with higher Mg bioavailability than Mg sulfate, which is an inorganic salt [38]. The superior bioavailability of Mg from citrate was also confirmed by results from our previous studies conducted on six Mg compounds administered to mice [39]. The selection of the three Mg compounds included in the study was based on results on Mg bioavailability reported by various researchers, as well as by our research team. The superior bioavailability of Mg from the Mg orotate included in the lipogel formulation confirmed once again that Mg^2+^ can cross the epithelial membrane in order to exert the therapeutic effect. The differences in the bioavailability of Mg from various compounds have also been studied by Ates et al., who reported higher bioavailability of Mg from organic compounds than from inorganic salts. The compound to which Mg^2+^ is bound may influence its bioavailability across different tissues, as the associated compound can facilitate intracellular Mg^2+^ transport. Thus, not all organic Mg compounds exhibit the same bioavailability in tissues; for example, taurate increases Mg^2+^ levels in the brain but not in muscle tissue, at the same dose [40].

Yang et al. reported beneficial effects of Mg^2+^ on wound repair after repeated application of a hydrogel in a murine model. In epidermal keratinocytes, Mg^2+^ stimulates hyaluronic acid production through activation of glycogen synthase kinase 3. Additionally, within the epidermis, Mg^2+^ reduces Ca^2+^ influx and expedites re-epithelialization following tissue injury [41].

Low extracellular Mg levels decrease glucose uptake and glyceraldehyde-3-phosphate dehydrogenase activity, which leads to changes in pyruvic acid and carnosine levels [42,43]. The results of several studies suggest that Mg plays an important role in skeletal muscle function by modulating the IGF-1/PI3K/Akt pathway, thereby influencing anabolic and catabolic processes and preventing muscle atrophy [44]. The involvement of Mg in muscle regeneration processes is linked to the activation of the myogenic genes Myf5, Myod, and Myog [45]. Although Mg does not have a direct analgesic effect, it decreases central pain sensitivity by blocking NMDA receptors and Ca^2+^ influx into cells [46]. The analgesic effect observed after Mg administration is due to its muscle-relaxant and vasodilator properties, which also support its use to improve muscle performance in athletes [47]. Mg is involved in the activity of glycolytic enzymes, in energy metabolism, and in maintaining muscle function (muscle contractility) as a result of the formation of the complex with ATP [48]. The role of Mg in maintaining muscle function is explained by its involvement in protein synthesis, together with RNA during transcription by forming phosphodiester bonds and a rigid structure, with oxygen atoms and phosphate groups involved in the translation stage [49]. Muscle tissue regeneration after physical activities or various diseases can be hindered by inflammatory processes. Low Mg levels are correlated with increased values of TNF-α, IL-1β, and C-reactive protein [50,51]. At the skeletal muscle level, Mg influences muscle damage by involvement in NF-kB activity [52].

As a result of Mg participation in neuromuscular disorders, the effects of Mg on nocturnal cramps in the lower limbs were studied. A decrease in the intensity and frequency of these symptoms was observed following the administration of magnesium supplements, particularly in pregnant women [53,54,55].

The inclusion of various active ingredients in topical preparations has become increasingly common alternative in recent years as an adjunctive therapy in the treatment of various pathologies [56,57].

In our clinical study, all patients received tacrolimus as first-line immunosuppression. This is a potent immunosuppressant, but its most notable adverse effect is hypomagnesemia due to its impact on tubular reabsorption of magnesium. The study included 21 kidney transplant patients who presented with hypomagnesemia but were unable to tolerate oral magnesium supplementation.

The pathogenic mechanisms underlying post-transplant hypomagnesemia are complex. It is well known that of the daily dietary magnesium intake, only 30–50% is absorbed in the small and large intestines, through a compensatory mechanism in which absorption is inversely proportional to dietary magnesium content. The kidney plays a central role in maintaining magnesium homeostasis. Once filtered by the glomerulus, magnesium is predominantly reabsorbed in the thick ascending limb of the loop of Henle, with a smaller proportion reabsorbed in the proximal or distal convoluted tubule. In addition, a functional kidney has an adaptive capacity to manage magnesium excretion, meaning that in the context of magnesium deficiency, reabsorption increases, and conversely, it decreases when magnesium is abundant. This mechanism is disrupted in the context of tubular injury, regardless of the underlying cause [58].

The use of tacrolimus, regardless of whether it is in immediate-release or extended-release form, more frequently causes hypomagnesemia compared to cyclosporine [59]. Tacrolimus appears to induce urinary magnesium wasting through modulation of epidermal growth factor (EGF) expression and TRPM6 channels in the distal convoluted tubule [60].

Magnesium deficiency leads to a range of clinical manifestations, from mild symptoms related to neuromuscular hyperexcitability to severe neurological disturbances [61,62,63,64]. Although it occurs more frequently in the first year post-transplant, it may persist indefinitely for the entire duration of graft survival, often correlating with some degree of renal graft dysfunction.

The patients selected for participation in this study had a mean age of approximately 50 years, a history of kidney transplantation of over 13 years, and stable renal function, with a glomerular filtration rate above 60 mL/min/1.73 m^2^. The majority were male, most received a graft from a deceased brain donor, and only one-third were employed.

The mean serum hemoglobin was within normal limits, with a mild inflammatory syndrome. Serum calcium, an ion involved in neurotransmission and often associated with magnesium metabolism, was also within normal limits. New-onset diabetes after transplantation (NODAT), frequently linked to hypomagnesemia through multiple pathogenic mechanisms, occurred in over one-third of these patients, reaching a level approaching statistical significance compared to patients without hypomagnesemia [65,66,67]. Post-transplant hypomagnesemia in renal transplant recipients drives insulin resistance, promotes atherogenic dyslipidemia, and negatively impacts cardiovascular pathology, the leading cause of post-transplant mortality [68,69,70]. Consistent with published studies, cardiovascular pathology was significantly more prevalent in these patients, and infections at various sites requiring hospitalization were also substantially more frequent [71,72].

Numerous studies have investigated the relationship between serum magnesium concentrations and muscle performance, neuropathic pain, or psychological status [73,74]. However, the findings have been inconsistent, partly due to differences in study design and partly because serum magnesium concentrations may not accurately reflect total body magnesium stores. Dominguez et al. (2006), for example, reported a positive association between serum magnesium levels and muscle performance in older adults [75].

The effects of magnesium supplementation on muscle performance have also varied across studies, depending on the study design, magnesium dose, and duration of supplementation. Positive findings have been reported in cross-sectional and non-placebo-controlled studies evaluating the potential association between a magnesium-rich diet and muscle performance [76,77]. In contrast, some randomized controlled trials have shown that low doses of magnesium supplementation (<250 mg/day) do not significantly influence muscle performance, whereas higher doses (>300 mg/day), administered for 5–12 weeks, have been associated with improvements in muscle performance [78,79,80,81].

In the present study, although serum magnesium increased significantly following treatment, the absolute increase was modest and did not restore magnesium concentrations to the normal reference range. The clinical relevance of this change is therefore difficult to determine, particularly given the complex regulation of magnesium homeostasis. Serum magnesium concentrations are influenced by multiple factors, including dietary intake, intestinal absorption, renal and extrarenal elimination, and intracellular magnesium distribution, several of which were not assessed in the present study. Consequently, the observed change in serum magnesium should be interpreted cautiously and cannot, by itself, be considered evidence of complete magnesium repletion or of a clinically meaningful improvement in neuromuscular function.

The evaluation of exercise capacity in patients with hypomagnesemia compared to normomagnesemic controls—groups that did not differ significantly in demographic or laboratory parameters—using questionnaires and quantified results, revealed an absence of high-intensity physical performance in the hypomagnesemic group, along with a significant reduction in moderate-intensity exercise capacity. In this context, we considered it useful to test the efficacy and safety of a magnesium orotate lipogel, applied twice daily to the limbs for 60 days. Although serum magnesium increased significantly following treatment, the absolute increase was modest and did not restore magnesium concentrations to the normal reference range. Thus, the observed change should be interpreted as a preliminary biological effect rather than as definitive correction of magnesium deficiency. Whether such a magnitude of change is sufficient to produce clinically meaningful improvements in neuromuscular function remains uncertain and warrants confirmation in larger, controlled studies using validated measures of neuromuscular outcomes. The lipogel administration proved safe, with no adverse effects on either renal function or inflammatory status, as evaluated by CRP levels.

The effects of the lipogel application on the limbs were assessed through exercise tolerance quantified in METs. Following topical administration of magnesium orotate, tolerance to moderate-intensity exercise increased significantly, while intolerance to high-intensity effort persisted. In the proposed linear regression model, the improvement in moderate-intensity activity was correlated with the increase in serum magnesium levels. Regarding patient satisfaction with the application of our product, no notable adverse effects were reported. Efficacy appeared higher in patients with greater social integration and those employed, with surprisingly better scores observed among male patients. The lipogel application did not interfere with daily activities, and the collaboration with both the pharmacist and the monitoring physician was highly positive, with satisfaction scores approaching the maximum possible rating.

The strengths of this study lie in the application of this magnesium therapeutic form to a population deficient in this ion and with no other viable therapeutic options, often with comorbidities potentially associated with hypomagnesemia. The study was interventional, pilot, before–and–after, non-randomized, and closely monitored, targeting a population with multiple associated risk factors. The demonstrated efficacy and tolerability of lipogel in this niche group encourage further evaluation in larger, more representative cohorts and support validation of the product at a population level.

The novel elements of this study are the comparative analysis of the degree of membrane penetration of the three Mg compounds included in the topical formulation, the identification of Mg orotate as the compound with a high level of permeation (97.84%), but also the complex formulation of a Mg lipogel that presents a high degree of acceptability, efficacy, practicability and lack of side effects. This aspect is all the more important given that many topical preparations containing various Mg compounds have not had their therapeutic efficacy demonstrated prior to being marketed, especially online. In this study, we included Mg compounds for which we conducted bioavailability studies in laboratory animals (administered internally) and that are also used in various topical preparations that are mainly marketed online. Magnesium orotate has the ability to penetrate cell membranes thanks to orotic acid which acts as a carrier, facilitating the penetration of magnesium into the cell and thus ensuring a superior bioavailability compared to other magnesium compounds. The two orotic acid molecules neutralize the positive charge of the magnesium ion and thus ensure the neutrality of this compound, while also giving it an increased affinity for the lipids in the cell membrane, which it crosses by passive diffusion, without energy consumption [82,83]. The lipogel base has an occlusive effect. It blocks the evaporation of water from the skin, causing a local hyperhydration of the stratum corneum that increases the metabolic activity of keratinocytes. As a result, transporters such as TRPM7 are stimulated to take up magnesium orotate much more efficiently. Magnesium contributes to increasing the number of AQP-3 channels (Aquaporin 3), increasing the internal flow of water and endogenous glycerol. The mechanism of absorption of magnesium through the skin is complex and involves passive transport mechanisms, transcellular pathways through the keratin layer but also through the sebaceous and sweat ducts. The lipid matrix acts as a reservoir that gradually releases magnesium ions through the skin layers following hydration and diffusion processes. Unlike classic magnesium oils that leave a sticky film and cause itching (due to the high concentration of chloride ionic salt), lipogel is a biocompatible structure. The lipid components are compatible with the skin’s sebum, incorporate magnesium orotate and help it cross the skin inwards, to the receptors. The lipogel applied by massage on the calves creates a micro-deposit in the hair follicles and upper layers of the skin. It releases magnesium slowly and steadily, ensuring muscle protection over a long period of time.

Magnesium orotate is insoluble and is incorporated in the form of a suspension. The combination of emollient, accelerated penetration and thermal effect provides a rapid and long-lasting action on the muscle tissue. Propylene glycol and water maintain it in a microdispersed state, while shea butter and eucerin form a protective film on the skin (depot effect). Magnesium migrates slowly from the carbomer and xanthan gum matrix, reaching the muscle tissue within a few hours.

The absorption of magnesium orotate from this specific lipogel formula is a complex, multiphase process, optimized by the presence of penetration promoters and the polymer matrix. Propylene glycol penetrates deep into the intercellular spaces, modifying the keratin structure and promoting the absorption of magnesium orotate at the epidermis level. Ethanol temporarily fluidizes the lipids in the skin and accelerates the diffusion of magnesium orotate inward. In addition to the cooling effect, menthol is a recognized penetration promoter. It disrupts the intercellular lipid order in the skin and thus promotes the penetration of magnesium orotate into the deep layers of the skin. The composition of the lipogel promotes the absorption of magnesium orotate, which crosses the epithelial barrier through the hair follicles and sebaceous glands but also through a slower intercellular route (depot). The matrix made up of carbomer 940, xanthan gum and lipids (shea, eucerin) retains some of the magnesium on the surface, gradually releasing it over a few hours. Once it passes through the epidermis and reaches the dermis (vascularized area), the major advantage of orotic acid comes into play. Skeletal muscle cells (affected by cramps) have specific transport systems for orotates. Unlike magnesium chloride or sulfate (which are recognized as simple inorganic salts and diffuse poorly through cell membranes), orotate is recognized by the cell as an essential nutrient (precursor of RNA and ATP). The cell membrane allows the magnesium orotate complex to pass inside the muscle cell. Once in the cytoplasm, the complex dissociates, releasing the magnesium ion exactly where it is needed to block calcium and stop the spasm. The lipogel formulation acts in two stages: rapid initial absorption (15–30 min) facilitated by ethanol and menthol which provides immediate pain relief and partial relaxation; and the second stage involves long-term absorption (2–6 h) supported by the hydro-lipid base, to prevent the recurrence of cramps (for example, during the night). The results of our study contribute to the correct choice of topical preparations with therapeutic efficacy. The use of standardized questionnaires to assess patient satisfaction contributes to the validity of the results obtained and supports the selection of the most effective strategies for alleviating neuromuscular symptoms, thereby promoting improved quality of life and enhanced occupational functioning.

This study has several limitations that should be acknowledged. First, it was a before-and-after, self-controlled pilot study without a placebo or standard-therapy control group. This design was chosen primarily because of the limited number of eligible participants and the study’s exploratory nature. Introducing a placebo-controlled arm would have further reduced the number of patients available for analysis and, consequently, the statistical power. Nevertheless, the absence of a control group limits the ability to distinguish the specific effects of the topical magnesium formulation from other factors that may have influenced the observed outcomes. In particular, a Hawthorne effect, subjective expectations, regression to the mean, and natural fluctuations in the clinical condition over time cannot be excluded. Therefore, the observed improvements in serum magnesium levels and physical activity should be interpreted cautiously, as a causal relationship with the intervention cannot be definitively established. Second, the relatively small sample size limits the study’s statistical power, increases the risk of type II error, and may make the results more sensitive to individual variability and outlying observations. Although the treatment and comparison groups were broadly similar in demographic and paraclinical characteristics, the limited sample size reduces the precision and stability of the estimates and restricts the generalizability of the findings. Accordingly, the results should be considered preliminary and hypothesis-generating rather than confirmatory. Third, as the study was exploratory, it was not designed to characterize the pharmacokinetic profile or systemic bioavailability of topical magnesium. Serial pharmacokinetic sampling and assessment of intravenous permeation testing (IVPT), maximum plasma concentration (Cmax), time to maximum concentration (Tmax), elimination half-life (t1/2), and area under the concentration–time curve (AUC) were therefore beyond the scope of the present investigation. Dedicated pharmacokinetic and bioavailability studies will be required to characterize systemic exposure, absorption, and the pharmacokinetic behavior of the topical formulation. Fourth, the IPAQ-SF is a self-reported instrument, and recall, reporting, and psychosocial factors may influence the accuracy of the results. As this was an exploratory study aimed at obtaining preliminary clinical data, the IPAQ-SF was used as a feasible assessment tool.. Fifth, immunosuppressive medication can produce certain changes in the skin, making it thinner and more fragile, and thus more permeable. Finally, the lack of randomization and the short-term, pre–post design further limit the ability to establish the durability of the observed effects and their independence from time-related changes. Larger, randomized, placebo-controlled studies with longer follow-up are warranted to confirm these preliminary findings, quantify the magnitude of the treatment effect, and establish the clinical efficacy and systemic exposure associated with the topical magnesium formulation.

## 5. Conclusions

All three Mg lipogel formulations presented appropriate organoleptic characteristics, good viscosity and adhesion to the skin, and pH 5.5, without exerting an irritating action on the skin. Regarding the diffusion of Mg through membranes, this was different; the highest release rate was achieved in the case of the Mg orotate lipogel (97.84%), followed by Mg citrate (62.74%) and Mg sulfate (35.05%). The degree of satisfaction of the 51 patients included in the study was high for all parameters monitored (practicability—86.6%, efficacy—96.4%, lack of adverse reactions—97.06%, trust in medical professionals—97.55%).

At present, there are no conclusive studies regarding the impact of topical Mg preparations on chronic disorders affecting skeletal muscle. However, the beneficial effects of Mg have been reported in neuromuscular diseases. In this context, the development of a Mg-containing pharmaceutical preparation for topical application can be regarded as a positive approach. Such a formulation may alleviate symptoms in patients with these conditions and may also limit the excessive use of orally administered medication. Moreover, the selected excipients allow not only the therapeutic action of Mg to occur in deeper tissues but also provide emollient and moisturizing effects on the skin at the site of application.

## Figures and Tables

**Table 1:** Lipogel composition prepared with Mg compounds.

Components	Formula 1 (g)	Formula 2 (g)	Formula 3 (g)	Role in Formulation
Magnesium sulphate	5.00	-	-	Therapeutic agent
Magnesium orotate	-	5.00	-	Therapeutic agent
Magnesium citrate	-	-	5.00	Therapeutic agent
Carbomer 940	1.00	1.00	1.00	Thickening agent
Propylene glycol	3.00	3.00	3.00	Polar permeation promoter
Triethanolamine	1.00	1.00	1.00	Neutralization agent
Xanthan gum	2.00	2.00	2.00	Thickening agent
Menthol	1.00	1.00	1.00	Absorption promoter
Diluted ethanol	5.00	5.00	5.00	Permeation promoter
Olive squalene	3.00	3.00	3.00	Penetration promoter
Hydrous Eucerin	6.00	6.00	6.00	Lipophilic excipient with emulsifying properties
*Butyrospermum Parkii* (Shea) Butter (shea butter derived from the shea tree)	1.00	1.00	1.00	Linear permeation promoter
Cosgard^®^ (Benzyl alcohol, Salicylic acid, Glycerin, Sorbic acid)	1.00	1.00	1.00	Antimicrobial agent
Purified water	71.00	71.00	71.00	Hydrophilic excipient

**Table 2:** Texture analyzer parameters for evaluating firmness, consistency, adhesiveness, and spreading capacity.

Texture Profile Analysis Parameters	Target Value (mm)	Trigger Load (g)
Firmness (g)	25	10
Consistency (mJ)	25	10
Adhesiveness (mJ)	25	10
Spreading capacity (g)	10	2

**Table 3:** Results of organoleptic testing.

Lipogels	Aspect	Color	Odor
Formula 1	Homogeneous	White–pearly white	Characteristic (menthol)
Formula 2	Homogeneous	White	Characteristic (menthol)
Formula 3	Homogeneous	White–pearly white	Characteristic (menthol)
Lipogels	Aspect	Color	Odor

**Table 4:** Characterization of lipogels based on textural parameters.

Texture Parameters (Mean ± SD)	Formula 1	Formula 2	Formula 3
Viscosity (cP)	145,200 ± 3135.7	144,600 ± 5414.2	145,850 ± 2578.5
Firmness (g)	48.65 ± 9.5	45.98 ± 6.2	47.92 ± 4.6
Consistency (mJ)	614.2 ± 7.6	610.5 ± 3.8	611.4 ± 8.2
Adhesiveness (mJ)	17.5 ± 1.3	18.1 ± 6.4	17.95 ± 5.4
Spreading capacity (mm^2^)	75.8 ± 2.1	73.5 ± 0.4	75.4 ± 6.1

**Table 5:** The percentage of Mg that crossed the artificial membrane.

Time (h)	Orotate Mg (mg%)	Citrate Mg (mg%)	Sulfate Mg (mg%)
0.5	20.12	10.26	5.32
1	24.47	15.67	8.76
2	38.52	28.13	14.22
3	48.95	31.32	17.52
4	62.58	42.33	22.15
5	73.38	46.99	26.29
8	83.17	50.22	31.14
12	95.33	59.54	33.58
24	97.84	62.74	35.05

**Table 6:** The baseline characteristics of the Study Cohort.

Patient No	Age (y)	Sex (F)	Tx vintage (y)	Donor type (D)	Employed (y/n)	eGFR (ml/min)	Mg (mg/dL)	Hb (g//dL)	hsCRP (mg/L)	Comorbidities
1	45	F	17	D	1	30	1.37	11.7	2.8	DM; infections
2	64	M	12	D	1	49	1.4	15.9	18.9	DM; CVD
3	41	M	10	D	1	43	1.59	13	9.1	-
4	55	M	13	D	0	96	1.32	15.3	1.6	DM;
5	32	M	20	L	1	83	1.55	13.7	3	-
6	68	F	6	D	0	21	1.4	11.5	15	infections
7	58	M	9	D	1	98	1.59	13.3	1.7	infections
8	36	M	19	L	0	55	1.56	13.3	1	infections
9	48	M	14	D	0	59	1.47	14.6	2.3	-
10	42	F	16	D	0	23	1.45	12	2.3	-
11	44	F	7	D	1	90	1.47	12.7	1	-
12	58	F	7	D	0	27	1.6	11.5	7	Neoplasia; Infections; CVD.
13	63	M	19	L	0	80	1.3	15.7	2.3	-
14	62	F	19	D	0	69	1.54	13	2.3	infections
15	43	M	23	D	0	67	1.2	12.7	15	DM
16	56	M	12	D	0	51	1.53	15.4	3.5	neoplasia
17	46	M	4	D	0	102	1.36	12	4	-
18	54	M	24	L	0	76	1.34	14	5.9	DM; CVD
19	51	M	10	D	1	58	1.5	18	7.3	Infections; DM; CVD
20	63	M	12	D	0	42	1.36	12.3	10.5	DM; CVD
21	41	F	15	D	0	56	1.5	12.7	1.2	-
Mean ± SD%	50.952 ± 10.161	33.33%	13.714 ± 5.640	80.95%	33.3%	60.715 ± 24.948	1.448 ± 0.111	13.538 ± 1.715	5.605 ± 5.258	

**Table 7:** Evaluation of laboratory parameters before and after administration of the magnesium orotate lipogel.

Parameters	T0 M + SD Me/IQR	T2 M + SD Me/IQR	Shapiro–Wilk	*p* ^1^	ES
Mg	1.448 ± 0.111 1.47/0.18	1.554 ± 0.124 1.55/0.15	0.019	<0.001	−0.100
eGFR	60.715 ± 24.948	61.143 ± 25.19	0.071	1	0
Hs-CRP	5.605 ± 5.258	5.719 ± 4.824	0.082	0.181	−0.338
Hb	13.538 ± 1.715 13/2.3	13.552 ± 1.718 12.9/2.1	0.023	0.430	−0.216

^1^ Wilcoxon rank.

**Table 8:** Functional exercise capacity assessment before and after magnesium lipogel use.

	Study Group IPAQ at the Baseline	Study Group IPAQ After Magnesium Lipogel Application	*p* ^1^
IPAQ1	0	0	-
IPAQ2	0	0	-
IPAQ3	5.190 ± 0.873	5.190 ± 0.873	-
IPAQ4	279.286 ± 89.907	310.475 ± 92.505	<0.001
IPAQ5	5.714 ± 0.561	5.714 ± 0.561	-
IPAQ6	40 ± 10.488	58.810 ± 3.775	<0.001
IPAQ7	284.048 ± 87.029	256.429 ± 80.330	<0.001
MET	6764.9 ± 2719.09	7766.286 ± 2920.815	<0.001

^1^ Wilcoxon rank.

**Table 9:** Baseline characteristics of the study and control groups.

Parameter	Study Group N = 21	Control Group N = 44	*p*	Shapiro–Wilk
Age (y)	50.952 ± 10.161	50.068 ± 12.422	0.778 *	
Sex (M)	14 (66.66%)	25 (65.81%)	0.448	
Tx vintage (y)	13.714 ± 5.640	13.023 ± 4.791	0.623	0.094
Deceased donor	16 (76.19%)	33 (75%)	0.917	
Employed (y/n)	7 (33.33%)	25 (56.81%)	0.077	
Mg (mg/dL)	1.448 ± 0.111	1.791 ± 0.145	<0.001	0.005
Hemoglobin (g/dL)	13.538 ± 1.715	13.177 ± 1.617	0.412 *	0.659
eGFR (mL/min/1.73 m^2^)	60.715 ± 24.948	72.318 ± 28.88	0.165	0.046
Hs-CRP (mg/L)	5.605 ± 5.258	4.691 ± 3.790	0.715 ^#^	0.001
T-chol (mg/dL)	192.950 ± 63.695	202.860 ± 39.827	0.447 *	0.617
HDL-chol (mg/dL)	46.857 ± 11.306	49.314 ± 11.246	0.409 *	0.102
Triglycerides (mg/dL)	152.762 ± 81.253	141.114 ± 45.431	0.972 ^#^	0.001
Calcium (mg/dL)	9.190 ± 0.551	9.245 ± 0.672	0.748	0.217
T proteins (g/dL)	6.890 ± 0.348	7.04 ± 094	0.221 *	0.226
Uric acid (mg/dL)	6.510 ± 1.353	6.161 ± 1.292	0.255 ^#^	0.043
Severe infections	7 (33.33%)	10 (22.72%)	0.363	
Neoplastic	2 (9.52%)	3 (6.81%)	0.655	
CVD	5 (23.8%)	3 (6.81%)	0.051	
NODAT	7 (33.33%)	6 (13.63%)	0.063	

* *t*-test; ^#^ Mann–Whitney U.

**Table 10:** Comparative evaluation of exercise tolerance in normomagnesemic and hypomagnesemic patients.

	Study Group	Control Group	*p*
IPAQ1	0	0.114 ± 0.321	0.114 ^#^
IPAQ2	0	6.364 ± 18.311	0.114 ^#^
IPAQ3	5.190 ± 0.873	5.818 ± 0.390	0.001 ^#^
IPAQ4	279.286 ± 89.907	298.977 ± 71.150	0.350 ^#^
IPAQ5	5.714 ± 0.561	5.682 ± 0.471	0.601 ^#^
IPAQ6	40 ± 10.488	44.318 ± 15.004	0.279 ^#^
IPAQ7	284.048 ± 87.029	236.03 ± 46.086	0.005 *
MET	6764.9 ± 2719.09	7875.909 ± 2117.947	0.197 ^#^

* *t*-test; ^#^ Mann–Whitney U.

**Table 11:** Comparative evaluation of exercise tolerance in the study group following administration of magnesium orotate lipogel versus the control group.

	Study Group After Topical Mg Lipogel Usage	Patients with Normal Mg Level	*p*
IPAQ1	0	0.114 ± 0.321	0.114 ^#^
IPAQ2	0	6.364 ± 18.311	0.114 ^#^
IPAQ3	5.190 ± 0.873	5.818 ± 0.390	0.001 ^#^
IPAQ4	310.475 ± 92.505	298.977 ± 71.150	0.574 ^#^
IPAQ5	5.714 ± 0.561	5.682 ± 0.471	0.601 ^#^
IPAQ6	58.810 ± 3.775	44.318 ± 15.004	<0.001 ^#^
IPAQ7	256.429 ± 80.330	236.03 ± 46.086	0.198 *
MET	7766.286 ± 2920.815	7875.909 ± 2177.947	0.822 ^#^

* *t*-test; ^#^ Mann–Whitney U.

**Table 12:** Model Coefficients—∆MET.

**R**	**R^2^**	**AIC**	**F**	**df1**	**df2**	***p***
0.844	0.713	274.196	22.347	2	18	<0.001
**Predictor**	**Estimate**		**SE**	**95% Confidence Interval**		***p***
				**Lower**	**Upper**	
Intercept	695.110		56.132	577.180	813.040	<0.001
∆Mg	2920.425		438.818	1998.501	3842.348	<0.001
∆eGFR	−7.318		7.580	−23.243	8.608	0.347

**Table 13:** Satisfaction scores following lipogel application in the study group.

Main Scores	Entire Group	Employed	Unemployed	*p*	Male	Female
Side effects	4 21/21 (100%)	-	-	-	-	-
Efficacy	3.841 ± 0.227	3.905 ± 0.252	3.667 ± 0.346	0.132	3.667 ± 0.346	3.905 ± 0.252
practicability	3.873 ± 0.166	3.905 ± 0.163	3.857 ± 0.171	0.565	3.929 ± 0.142	3.762 ± 0.163
Everyday life	3.762 ± 0.319	3.810 ± 0.123	3.714 ± 0.316	0.498	3.667 ± 0.320	3.905 ± 0.252
Healthcare	4 21/21 (100%)	-	-	-	-	-
General satisfaction	4 21/21 (100%)	-	-	-	-	-
General score	3.894 ± 0.205	3.937 ± 0.081	3.873 ± 0.114	0.226	3.877 ± 0.111	3.929 ± 0.095

**Note:** All participants completed the questionnaire. Scores are reported according to the questionnaire’s scoring system, with 4 representing the maximum score. All participants (21/21; 100%) reported no adverse effects and assigned the maximum score of 4 for “Side effects”; the “Healthcare” and “General satisfaction” items were also rated 4 by all participants.

## Data Availability

The data presented in this study are available upon request from the corresponding author. The data are not publicly available due to ethical reasons.

## Supplementary Materials

The following supporting information can be downloaded at https://www.mdpi.com/article/10.3390/nu18162740/s1. Table S1. The Lübeck medication satisfaction questionnaire.

## Abbreviations

The following abbreviations are used in this manuscript:

IPAQ	International Physical Activity Questionnaire
MET	metabolic equivalent task
Mg	Magnesium
ATP	adenosine triphosphate
SD	standard deviation
eGFR	estimated glomerular filtration rate
hs-CRP	high-sensitivity C-reactive protein
CRP	C-reactive protein
AC06028	Abbott Alinity c analyzer AC06028
CMIA	chemiluminescent microparticle immunoassay CMIA
HQ00687	Abbott Alinity hq analyzer HQ00687
IPAQ-SF	International Physical Activity Questionnaire—Short Form IPAQ-SF
KMO	Kaiser–Meyer–Olkin
MET-min/week	total weekly physical activity
ΔMg	serum magnesium
ΔeGFR	renal function
ΔMET	exercise capacity
EGF	epidermal growth factor
NODAT	New-onset diabetes after transplantation
