# Supplementary material for: Topical Magnesium Orotate Lipogel in Kidney Transplant Recipients with Persistent Hypomagnesemia: Formulation Development and Pilot Clinical Study
Source: Nutrients. 2026 Aug 21;18(16):2740. doi: 10.3390/nu18162740 (PMC13516101; doi:10.3390/nu18162740)
Supplement: Supplementary file 1 [file nutrients-18-02740-s001.zip › nutrients-4485830-supplementary.pdf]

Topical Magnesium Orotate Lipogel in Kidney Transplant Recipients with Persistent Hypomagnesemia: Formulation Development and Pilot Clinical Study

Corina Moisa [1,†], Florin Bănică [1,†], Ioana Adela Rațiu [1,*], Octavia Gligor [1], Laura Grațiela Vicaș [1,*], Cristian Adrian Rațiu [1], Mădălin Florin Ganea [1], Csaba Nagy [2], Anamaria Ratiu [3], Edy Hagi Islai [3] and Mariana Ganea [1]

[1] Faculty of Medicine and Pharmacy, University of Oradea, 1st December Square 10, 410073 Oradea, Romania; cmoisa@uoradea.ro (C.M.), 0000-0001-9918-250X; fbanica@uoradea.ro (F.B.), 0000-0001-5998-9157; octavia.gligor@uoradea.ro (O.G.), 0000-0002-6672-1535; cristian.ratiu@didactic.uoradea.ro (C.A.R.), 0000-0002-1862-8758; ganea.madalinflorin@student.uoradea.ro (M.F.G.), 0009-0006-6719-5537; mganea@uoradea.ro (M.G.), 0000-0003-4643-2151
[2] Doctoral School of Biomedical Sciences, University of Oradea, 1 Universității Street, 410073 Oradea, Romania; nagy.csaba@student.uoradea.ro, 0009-0008-2375-9884
[3] Faculty of Dentistry, University of Medicine and Pharmacy "Iuliu Hatieganu" Cluj-Napoca, Victor Babeș Street 8, 400347 Cluj-Napoca, Romania; ratiu.anamaria@elearn.umfcluj.ro (A.R.); hagi.islai.edy@elearn.umfcluj.ro (E.H.I.)
* Correspondence: ioana.ratiu@didactic.uoradea.ro (I.A.R.), 0000-0003-0879-1903; lvicas@uoradea.ro (L.G.V.), 0000-0001-5328-333X
† These authors contributed equally to this work.

2. Materials and Methods

2.2. Phase II: In Vivo Evaluation of the Mg Orotate-Containing Lipogel Effectiveness

2.2.2. Data Collection

Table. S1 The Lübeck medication satisfaction questionnaire

	Statement	1	2	3	4
LMSQ_1	My medication schedule suits me well.				
LMSQ_2	I feel restricted in my daily activities because of the side effects of my medications.				
LMSQ_3	My medication is very convenient to take.				
LMSQ_4	Overall, I am satisfied with my treatment.				
LMSQ_5	My symptoms are improved by my medication.				
LMSQ_6	I feel that the doctor and pharmacist inform me properly about my illness.				
LMSQ_7	I am satisfied with the characteristics of my medications.				
LMSQ_8	The advantages and disadvantages of the treatment options were explained to me in detail by the physician and pharmacist				

	Statement	1	2	3	4
LMSQ_9	I am not able to do as much physical activity as I used to because of the side effects of my medications.				
LMSQ_10	My medication helps me perform personal hygiene tasks (brushing my teeth, showering, etc.).				
LMSQ_11	Prior to the treatment, I felt worse than I do now.				
LMSQ_12	The medication helps me get through everyday life.				
LMSQ_13	The doctor and pharmacist informed me about the best treatment option.				
LMSQ_14	I am content with the time it takes for my medication to start working.				
LMSQ_15	I am satisfied with my treatment.				
LMSQ_16	Thanks to the medication, I can participate in recreational activities.				
LMSQ_17	I cannot enjoy my free time as much anymore because of the side effects of my medications				
LMSQ_18	I intend to continue my treatment.				
